# Recent Bio-Advances in Metal-Organic Frameworks

**DOI:** 10.3390/molecules25061291

**Published:** 2020-03-12

**Authors:** Isobel Tibbetts, George E. Kostakis

**Affiliations:** Department of Chemistry, School of Life Sciences, University of Sussex, Brighton BN1 9QJ, UK; it67@sussex.ac.uk

**Keywords:** bio metal-organic frameworks (bioMOFs), drug delivery, imaging, catalysis, bioinspired

## Abstract

Metal-organic frameworks (MOFs) have found uses in adsorption, catalysis, gas storage and other industrial applications. Metal Biomolecule Frameworks (bioMOFs) represent an overlap between inorganic, material and medicinal sciences, utilising the porous frameworks for biologically relevant purposes. This review details advances in bioMOFs, looking at the synthesis, properties and applications of both bioinspired materials and MOFs used for bioapplications, such as drug delivery, imaging and catalysis, with a focus on examples from the last five years.

## 1. Introduction 

Metal-organic frameworks (MOFs) are a type of porous coordination polymer (CP), consisting of metal ions or clusters coordinated to organic linkers. Alkali and transition metals, lanthanides and actinides are all used in the construction of MOFs, with different metals adopting alternative coordination geometries or having potentially advantageous luminescent or fluorescent properties [1]. The first MOF was reported in 1989 when Robson et al. reported a ‘*framework consisting of three-dimensionally linked rod-like segments*’ [2]. The term MOF was introduced in 1995 [3], and the field began to gain momentum in the late 1990s. In 1999, Williams et al. published on a MOF known as HKUST-1, made from copper-based clusters and benzene tricarboxylate linkers [4]. This was followed by Yaghi et al. who reported a MOF known as MOF-5, made from zinc-based clusters and benzene dicarboxylate linkers, later in the year [5], which broke records with its extremely high porosity.

Unlike other porous materials, the internal surface chemistry of MOFs can be tuned, allowing properties such as hydrophilicity and acidity to be altered as desired [6]. However, the defining feature of MOFs is their ultrahigh porosity (up to 90% free volume) and large internal surface areas (ISA), with some MOFs reported to have over 10,000 m^2^ g^−1^ ISA [7]. MOFs are commonly synthesised using hydro- or solvothermal reactions [7]. Recently, this has expanded into more environmentally friendly procedures, utilising microwave, flow or mechano techniques [8,9,10]. In terms of the tuneable structures of MOFs, avoiding interpenetration—where multiple lattices can become entwined and affect the pores of the MOF—may be required when pore volume is the key factor [11]. Additionally, defects can be engineered in to provide additional adsorption or catalytic sites [12]. However, the functionalisation of the framework can alter the properties of a MOF most drastically. For example, catalysts could feature the active site at the ligand, at the metal node, or as a guest embedded within the pores [13]. Metal leaching has been observed in some cases, which could diminish catalytic activity, disrupt the MOF framework or limit use in vivo due to toxicity concerns [14].

These properties make MOFs attractive options for purposes such as fuel gas storage, the capture of greenhouse gases, and new catalytic procedures, in addition to improving upon the roles that zeolites previously fulfilled [15]. More recently, MOFs have also been used for biomedical purposes, such as exploiting the porosity of the material for drug delivery systems (DDS) [16]. Water stability and biocompatibility of MOFs is a particular issue in this area; degradation of the MOF or metal leaching could lead to unwanted toxicity, but water sensitivity could alternatively be advantageous; moisture was shown to trigger the release of encapsulated allyl isothiocyanate from a zinc-based MOF in 2016 [17]. The commercial viability of MOFs are evident, and a spin-off company, ‘MOFgen’, which utilises MOFs to release antimicrobial NO gas for medicinal purposes, was set up in 2013 [18,19]. Reportedly the first clinical trial involving MOFs was announced in 2018 [20], aiming to enhance the efficiency of X-ray radiotherapy (RT) as a cancer therapeutic. MOFs have been explored for applications such as drug delivery, biosensing, biocatalysis, imaging and as antimicrobial materials [21,22]. Toxicity issues can be addressed by the synthesis of Metal Biomolecule Frameworks, or bioMOFs, which incorporate biocompatible metals and active pharmaceutical ingredients (APIs) or endogenous molecules to form the porous materials [21].

**Figure 1:**
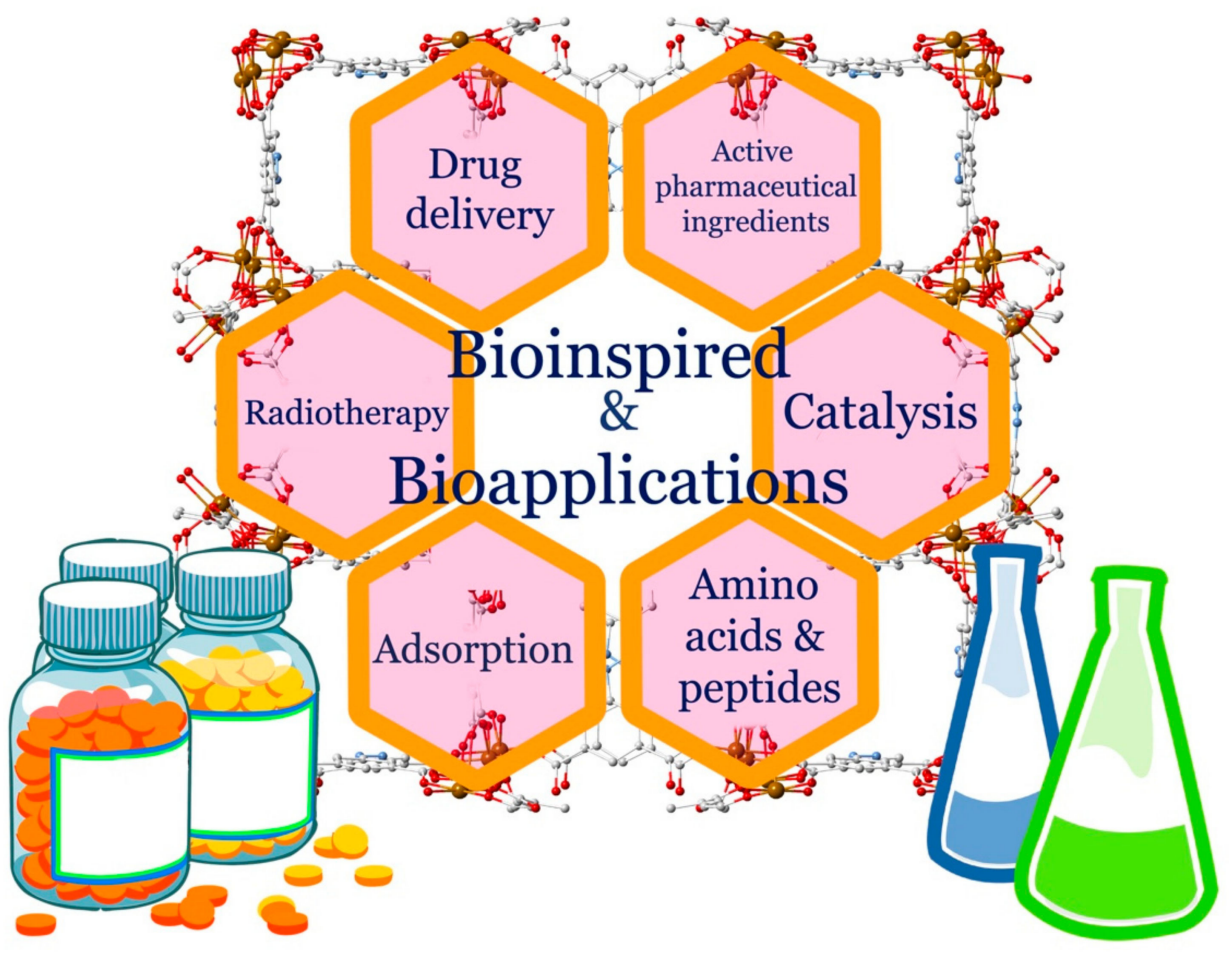
Schematic representation of bioapplications and biomaterials or active species used in the synthesis of metal-organic frameworks (MOFs).

The popularity of these materials for biomedical applications has been highlighted in recent reviews [22,23]. Chedid and Yassin explored recent examples of the use of MOFs and covalent organic frameworks (COFs) for biomedical applications, focusing on their synthesis and use as DDS [23]. Similar to MOFs, COFs have been used for energy and catalysis purposes [24,25], in addition to applications including biosensors and DDS [26]. More recently, Yang and Yang surveyed the current field of MOF-based biomedical materials, reporting advances in five areas including DDS, biosensing and biocatalysis [22]. Both reviews examined future challenges, such as regulatory difficulties and a need for further in vivo investigations. In addition to discussing these areas, this review explores applications, such as remediation of pharmaceutical waste, catalytic synthesis of API intermediates and the synthesis and applications of MOFs incorporating APIs or endogenous small molecules as part of their framework. As depicted in Figure 1, examples of both MOF bioapplications and bioinspired MOFs will be discussed and the prospects and challenges of the future of this field are explored.

## 2. Metal-Organic Frameworks (MOFs) Bioapplications

### 2.1. MOFs and Drug Delivery

The porous nature of MOFs can be used for a variety of host-guest chemistry, including drug delivery. This was first achieved in 2006 by Férey et al., who encapsulated isobutylphenylpropanoic acid (ibuprofen) in MIL-100 and -101; thus, forging the way for further work using MOFs as DDS [16]. Férey initially proposed MIL-100(Cr) be used for hydrogen storage [27]; more recently, MIL-100(Fe) nanoparticles have been coated in a lipid bilayer and used to store dye molecules [28]. It has also been used in cutaneous patches with biopolymers to deliver molecules including caffeine or ibuprofen [29]. Biocompatibility—the property of a material that is not harmful to living tissue—is an essential factor in the design and synthesis of MOFs that are to be used as a DDS in humans. The search for biocompatible MOFs has resulted in a shift from the use of metals, such as chromium in early MOFs, to non-toxic analogues, such as iron, zinc and zirconium [30]. MOFs have been used to encapsulate both small molecule inhibitors and larger, more complex molecules, such as hormones. In 2018, Farha et al. used a MOF, NU-1000, to develop a potential alternative formulation for insulin, with the framework protecting the hormone from degrading in the presence of stomach acid and pepsin, a digestive enzyme [31]. As DDS, MOFs can provide protective formulations [31], enhance the solubility of an API [32], allow slow release of a drug for a more stable pharmacokinetic profile [33], and act synergistically through the design of a bioactive cation and linker [34].

#### 2.1.1. Structure-Activity Relationship Between MOFs and Their Cargo: The Effect of Hydrophilicity/Hydrophobicity and Pore Size

**Figure 2:**
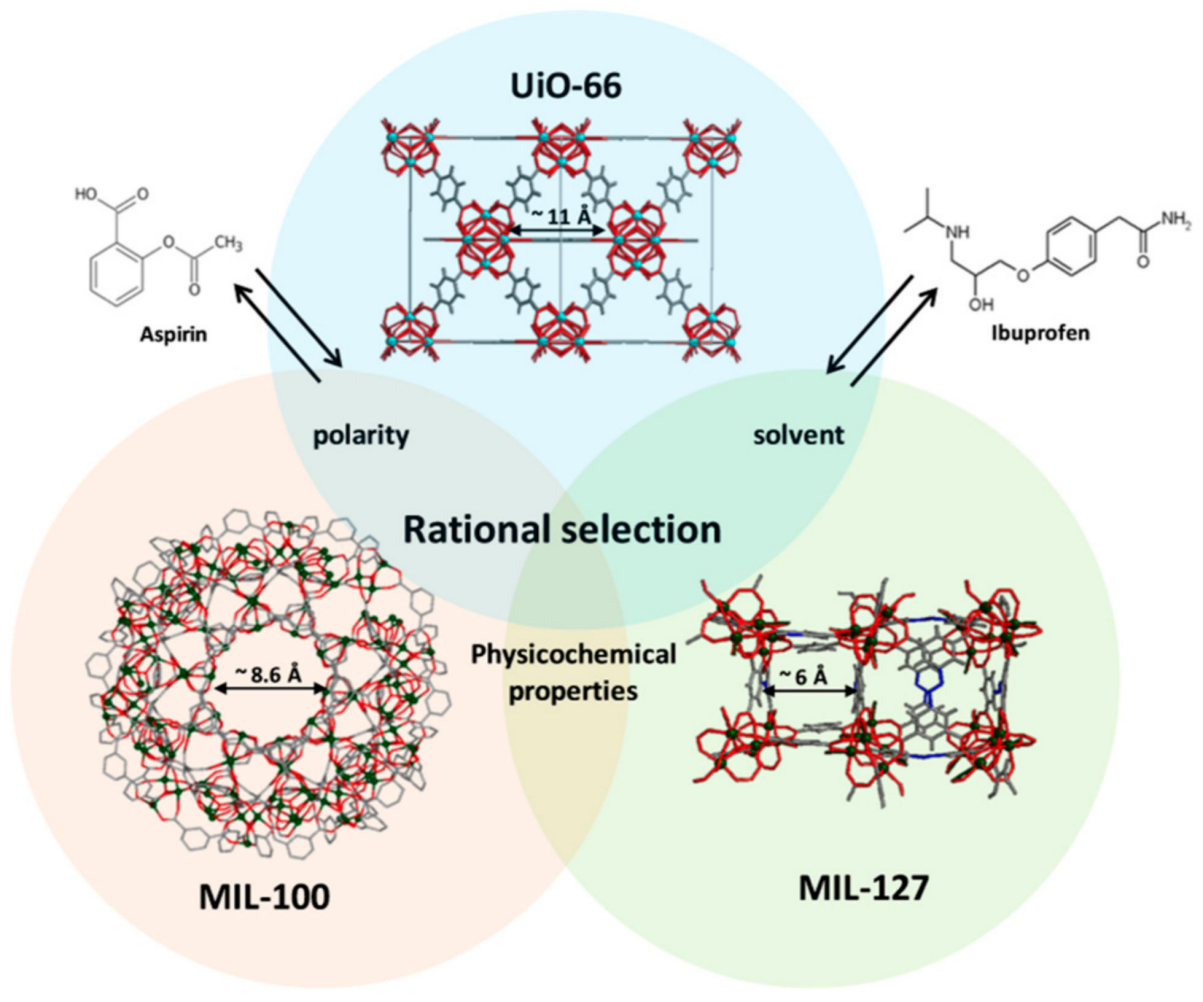
Structures of acetylsalicylic acid (aspirin) (left) and isobutylphenylpropanoic acid (ibuprofen) (right) with structural representation of UiO-66, MIL-100 and MIL-127. Zirconium polyhedra are cyan, iron polyhedra are green, nitrogen is blue, oxygen is red, and carbon is grey. Hydrogen atoms have been removed for clarity [33] Reprinted from S. Rojas, I. Colinet, D. Cunha, T. Hidalgo, F. Salles, C. Serre, N. Guillou and P. Horcajada, ACS Omega, 2018, 3, 2994–3003, available at https://pubs.acs.org/doi/10.1021/acsomega.8b00185. Any further permissions related to the material excerpted should be directed to the ACS.

Many MOF-drug delivery studies have focused on model drugs, such as ibuprofen, to achieve proof of concept. However, a more profound understanding is required to allow for a set of principles to be used in MOF DDS research and to allow for the encapsulation of a range of drugs with different properties. A comprehensive study by Horcajada and co-workers aimed to understand the effect of the features of both MOF and drug on the drug encapsulation and release processes [33]. The relationship between three MOFs—MIL-100 (Fe), MIL-127 (Fe) and UiO-66 (Zr)—and two drugs, ibuprofen and acetylsalicylic acid (aspirin), was studied, focusing on parameters such as hydrophilicity/hydrophobicity and pore size (Figure 2). Furthermore, cutaneous administration was studied, rather than intravenous which had been the focus of most MOF studies of this kind [35,36,37].

MOFs consist of polar metal ions and nonpolar organic ligands which typically results in an amphiphilic interior. This, combined with the large pores characteristic of MOFs, makes them well suited to the encapsulation of large molecules such as pharmaceuticals. There is a need to develop structure-activity relationship (SAR) data for MOFs and the drugs they encapsulate and to understand the process of drug incorporation and delivery concerning existing MOFs and their properties. Previous works have studied the effects of linker properties, MOF topology and polarity on the DDS process, but only more recently are researchers focusing on the nature of the drug and its impact on the drug delivery process [35,36,37].

**Scheme 1:**
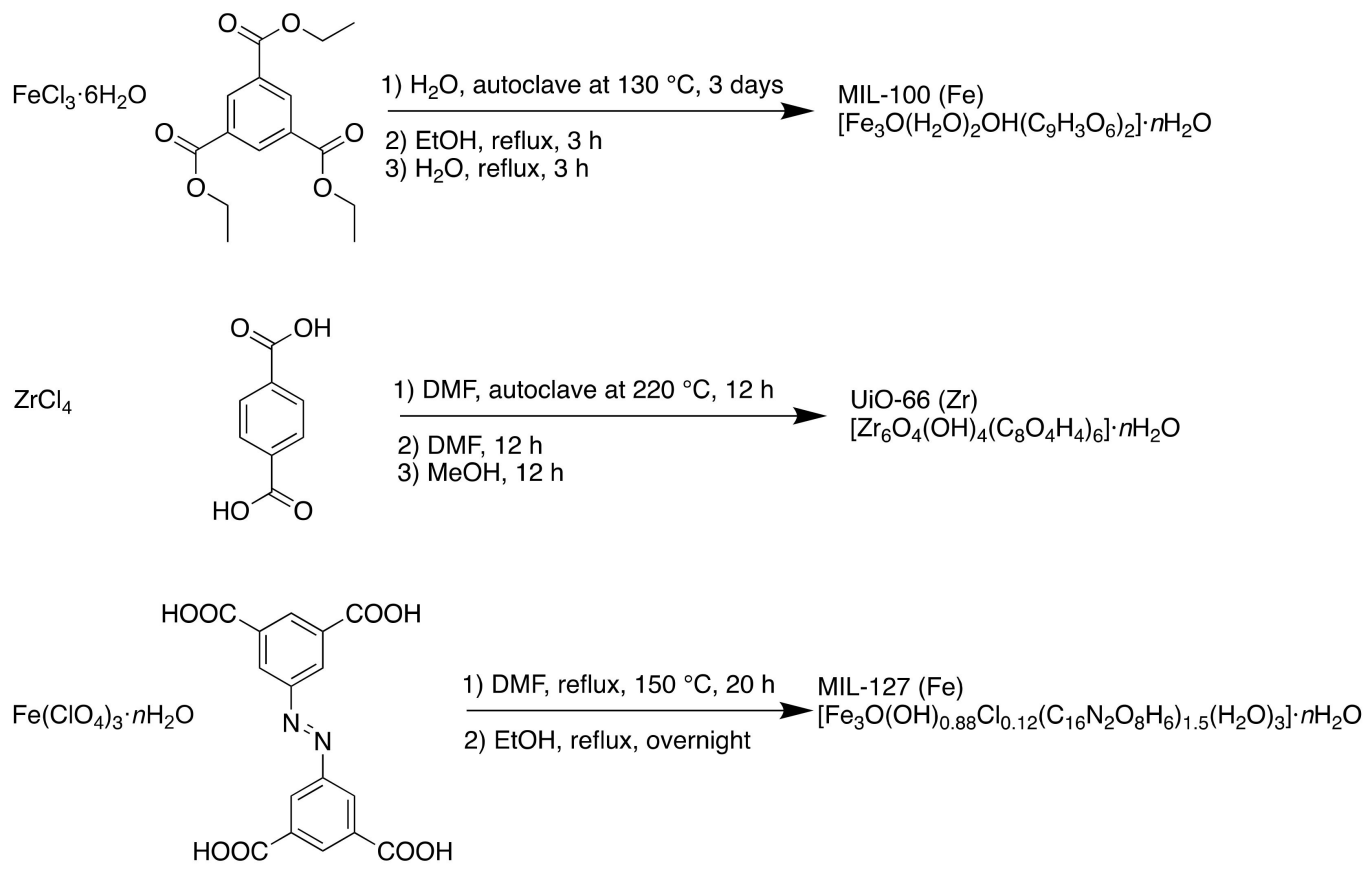
Synthesis of MIL-100 (Fe), UiO-66 (Zr) and MIL-127 (Fe) using solvothermal and reflux conditions.

The MOFs were synthesised using solvothermal and reflux methods as detailed in Scheme 1.

To mimic the cutaneous environment, the drug release experiments were performed in distilled water at 37 °C. It was found that the hydrophobicity/hydrophilicity of the drug and the accessibility of the MOF pores were the main parameters affecting the rate of drug delivery. This offers the potential to tailor the MOF to a chosen drug, depending on its properties. The properties of the MOFs are detailed in Table 1; fast delivery was seen with the more hydrophilic, mesoporous MIL-100, slow delivery was a result of the narrow pored, amphiphilic MIL-127, and the rate of delivery depended on the hydrophobicity or hydrophilicity of the drug in the case of the microporous, hydrophobic UiO-66. Aspirin has a logP of 1.19, is hydrophilic and was released quickly, while ibuprofen has a logP of 3.97—hydrophobic by comparison—and was released slowly from this MOF.

The suitability of MOFs to a cutaneous approach was also proved due to the high drug loading seen (up to 35.5 *wt* %) and aqueous stability, associated with the ability to release a drug within 1–7 days. It is clear that a focus on the physicochemical properties of the encapsulated drug is required when considering DDS, rather than solely that of the MOF.

#### 2.1.2. Sugar-Based MOFs: The Benefits of a Renewable, Soluble Material

**Scheme 2:**
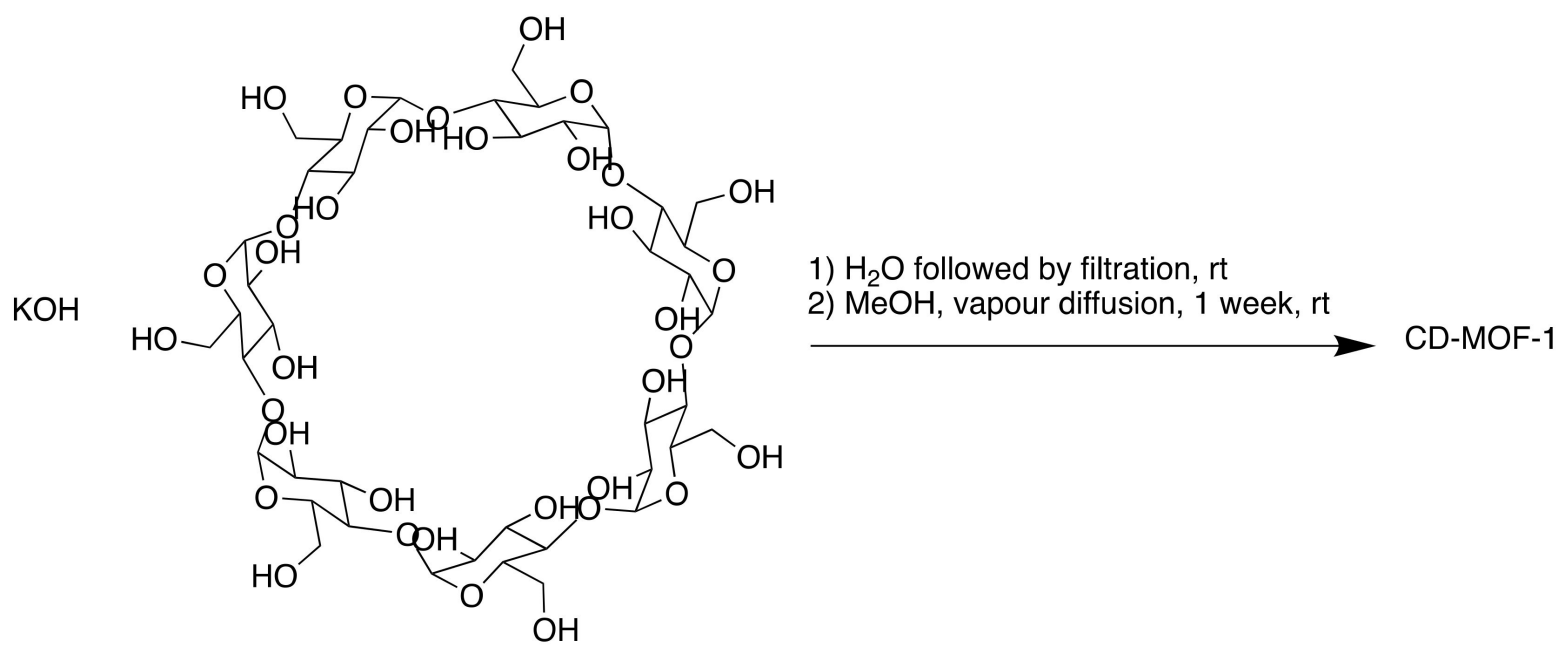
Synthesis of cyclodextrin (CD)-MOF-1.

In 2010, Stoddart et al. reported the synthesis of a MOF made from edible materials—namely, cyclodextrins (CDs) (Scheme 2) [38]. The MOF was made of renewable materials (CDs are produced from starch) and was water-soluble, the desired property in oral drugs.

Recently, this MOF has been used as a separation medium (2016) [39] and later, in a medicinal context—in 2019, CD-MOF was used to encapsulate folic acid (FA), used as a model molecule, achieving molar ratios of up to 2:1 FA:CD [32]. FA within CD-MOF demonstrated a 1450-fold increase in solubility compared to free FA, and in rats, the bioavailability of FA was increased by a factor of 1.48. 

**Figure 3:**
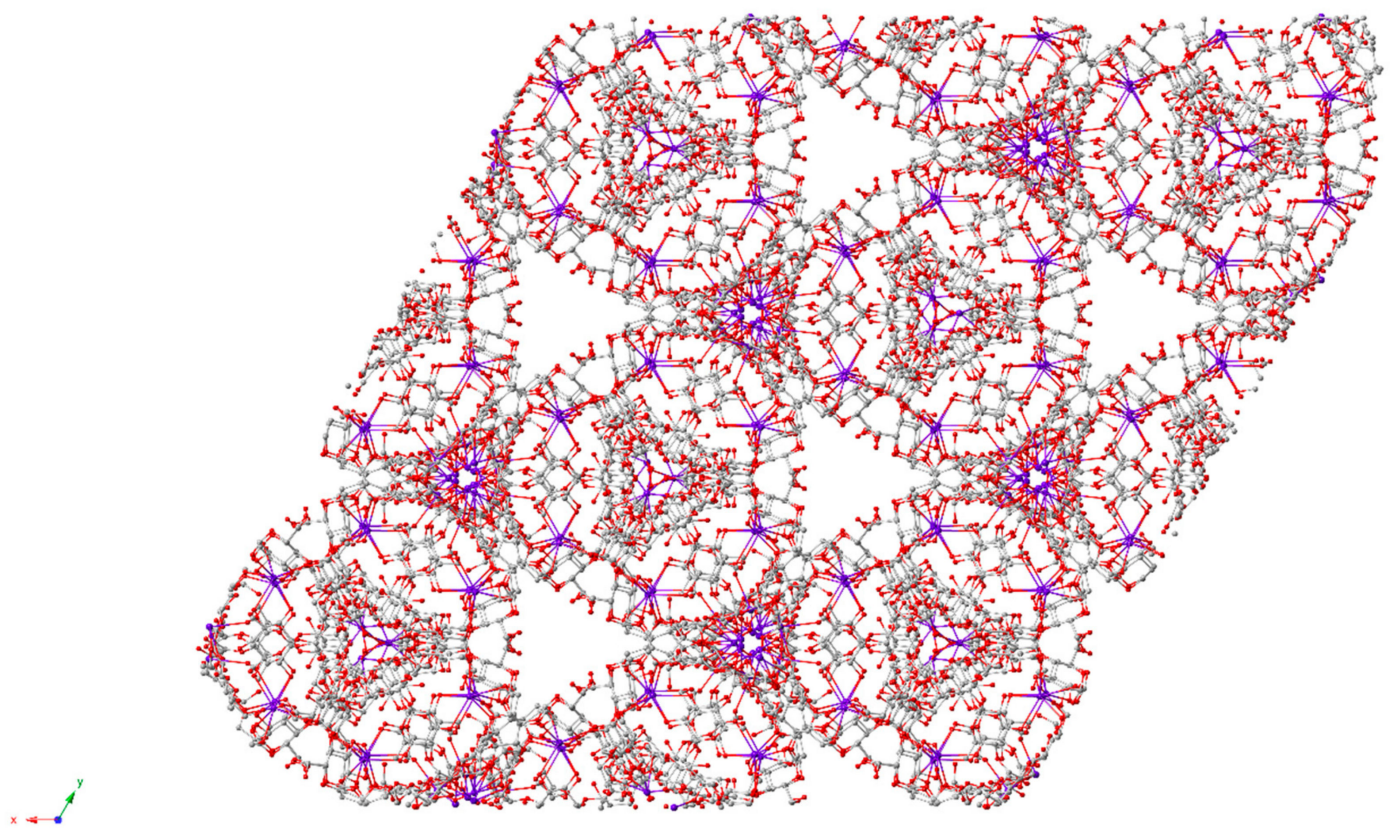
Space-filled representation of the extended solid-state structure of CD-MOF-1 where C is grey, O is red and K is purple [40].

CD-MOF has also been used to offer the potential for longer-lasting pain relief [40]. Ibuprofen, a commonly used painkiller, as a free acid, is poorly soluble in physiological environments. Therefore, increasing the half-life of the drug and its duration of action would be beneficial for patients. As cyclodextrins are known to improve the bioavailability of drugs by improving their solubility [41], CD-MOF (Figure 3) was perfectly suited to this application.

Incorporation of the drug was achieved using two methods; firstly, via crystallisation, using the potassium salt of ibuprofen as the source of the alkali metal cations required to form the MOF. This method utilises the fact that MOFs consist of a metal ion and an organic ligand—here, the salt of a drug, and a sugar. The incorporation of ibuprofen anions achieved charge balance. Secondly, the free acid form of ibuprofen was absorbed in a pre-synthesised MOF. In this case, potassium hydroxide was used as the alkali metal source. However, ibuprofen loading was similar in both cases (23 *wt* % vs. 26 *wt* %, respectively).

The potassium salt of ibuprofen is soluble in water, unlike the free acid form. Formulations of this type have faster uptake rates but are only available as gel capsules due to the hygroscopic nature of the potassium salt. Therefore, the ability of CD-MOF to encapsulate ibuprofen in pharmaceutically relevant quantities is valuable. The MOF demonstrated similar bioavailability and rapid blood plasma uptake to the pure potassium salt, showing that CD-MOF can act as an active delivery agent for ibuprofen. Moreover, when the drug was encapsulated in the sugar-based MOF, the duration of ibuprofen in blood plasma was doubled compared to ibuprofen salts alone.

CD-MOF-1 is nanoporous and has a Langmuir surface area of 1320m^2^·g^−1^ and an estimated total pore volume of 54% [38]. The cavity of cyclodextrin is hydrophobic, and in solution, they are known to host a variety of substrates [42]. The pore diameters in CD-MOF-1 range from 0.42 to 0.78 nm with a central pore of 1.7 nm in each (γ-CD)_6_ cube. These values are similar to the amphiphilic, microporous MIL-127 (Fe) from which slow delivery of ibuprofen was observed and reinforce the conclusions drawn by Horcajada et al. that MOF hydrophilicity/hydrophobicity and pore size play a large part in the rate of release of a drug [33].

Since Férey et al. first encapsulated ibuprofen in a MOF in 2006, ibuprofen has often been used as a ‘model drug’ in these studies. However, few have focused on oral administration or in vivo activity [40]. By utilising food-grade, soluble γ-cyclodextrins in the synthesis of this MOF, the effects of oral administration could be studied, a desirable property for a MOF DDS. There is clear potential for creating other CD-MOF-drug co-crystals and extending this to other therapeutic agents. The ease of synthesis of this non-toxic, biocompatible MOF make it an attractive option as a DDS.

#### 2.1.3. MOFs as Cancer Treatments: Radiotherapy and Transport of Reactive Oxygen Species

In addition to applications in drug encapsulation and delivery, MOFs have been used as radioenhancers, agents which sensitise tumour cells towards radiation therapy and are used to increase its effectiveness. Roentgen reported the discovery of X-rays in 1895, and radiotherapy has been used as a cancer treatment since soon after that discovery [43,44]. While an important therapeutic tool, side-effects including damage to neighbouring, healthy organs, has resulted in a search for compounds and techniques to enhance a tumour-targeting effect [45,46]. Heavy metal-based nanoparticles (NPs) have been studied for this purpose, as elements of high atomic number have corresponding high X-ray absorption coefficients [47]. Increased uptake by tumours leads to an increased radiosensitivity difference between the tumour and surrounding healthy tissue.

**Figure 4:**
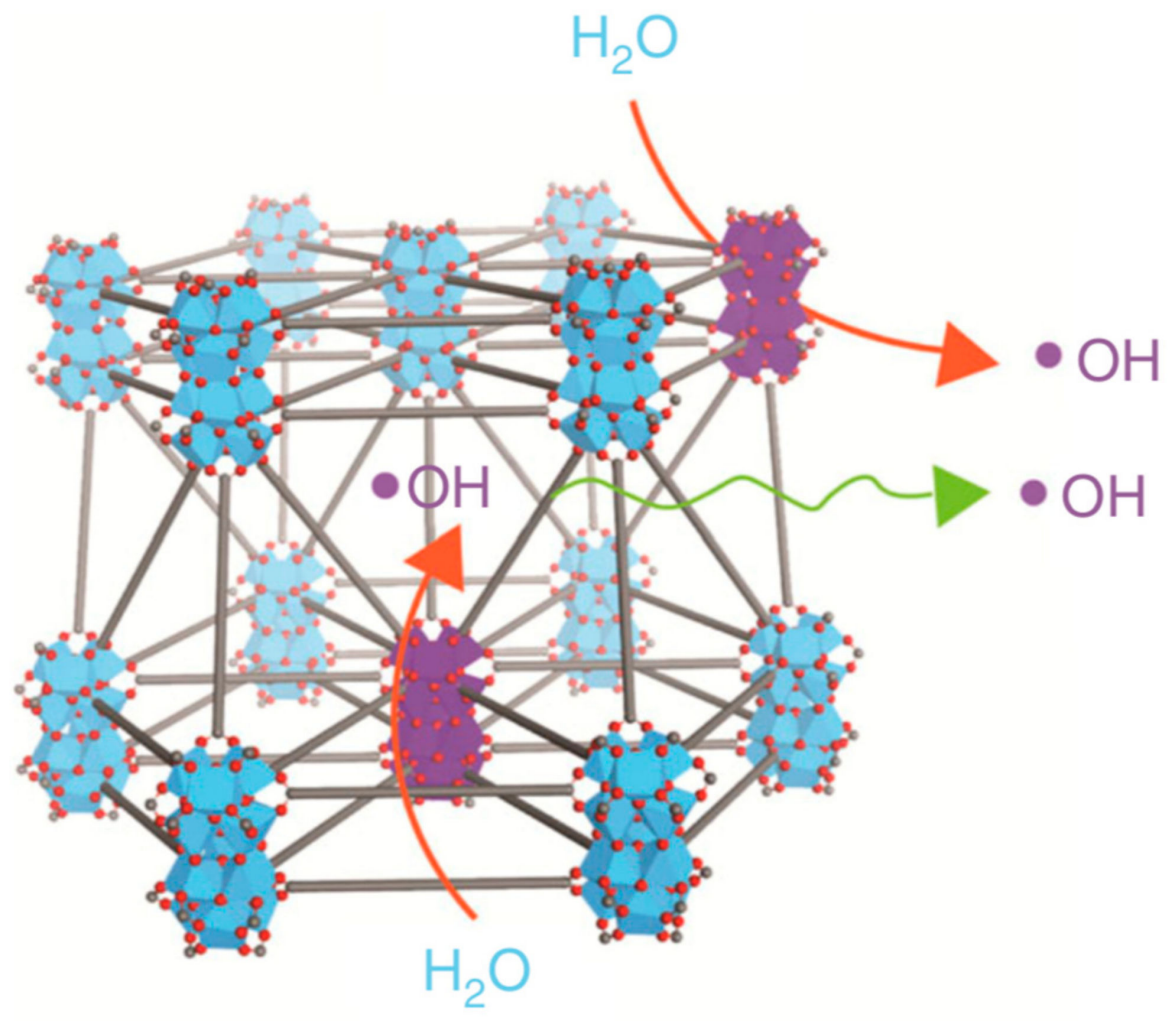
Representation of hydroxyl radical (ROS) generation upon X-ray irradiation and diffusion through Hf_12_-DBA. Adapted from [48] This article is licensed under a Creative Commons Attribution 4.0 International License, which permits use, sharing, adaptation, distribution and reproduction in any medium or format, as long as appropriate credit is given to the original author(s) and the source, a link to the Creative Commons license is provided, and changes made are indicated. http://creativecommons.org/licenses/by/4.0/ This image is a cropped version of the original figure in K. Ni, G. Lan, C. Chan, B. Quigley, K. Lu, T. Aung, N. Guo, P. La Riviere, R. R. Weichselbaum and W. Lin, Nat. Comms., 2018, 9, 2351.

Lin et al. theorised that the electron-dense secondary building units (SBUs)—inorganic polynuclear clusters—in nanoMOFs could preferentially absorb X-rays, thereby increasing radiosensitivity [48]. Additionally, the porous nature of the chosen MOF would allow the transport and diffusion of reactive oxygen species (ROS), used to exert a cytotoxic effect in photodynamic therapy (Figure 4). Furthermore, they hoped to combine nanoMOF radiotherapy with checkpoint blockade immunotherapy, a treatment which enhances antitumour immune system responses by targeting T cell inhibitory pathways.

**Figure 5:**
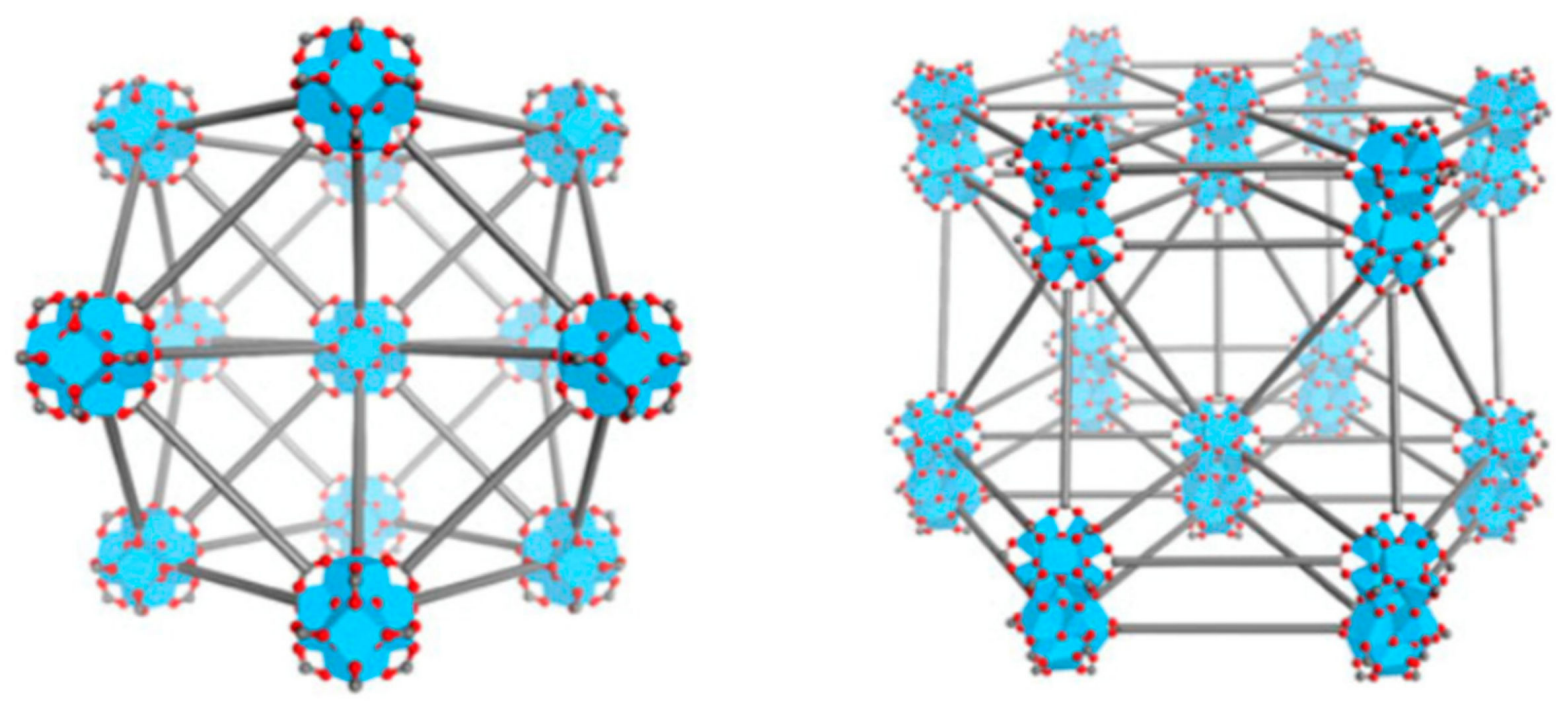
Structural representations of Hf_6_-DBA or Hf_6_-DBAn (left) and Hf_12_-DBA or Hf_12_-DBAn (right). Adapted from [48] This article is licensed under a Creative Commons Attribution 4.0 International License, which permits use, sharing, adaptation, distribution and reproduction in any medium or format, as long as appropriate credit is given to the original author(s) and the source, a link to the Creative Commons license is provided, and changes made are indicated. http://creativecommons.org/licenses/by/4.0/ This image is a cropped version of the original figure in K. Ni, G. Lan, C. Chan, B. Quigley, K. Lu, T. Aung, N. Guo, P. La Riviere, R. R. Weichselbaum and W. Lin, Nat. Comms., 2018, 9, 2351.

**Scheme 3:**
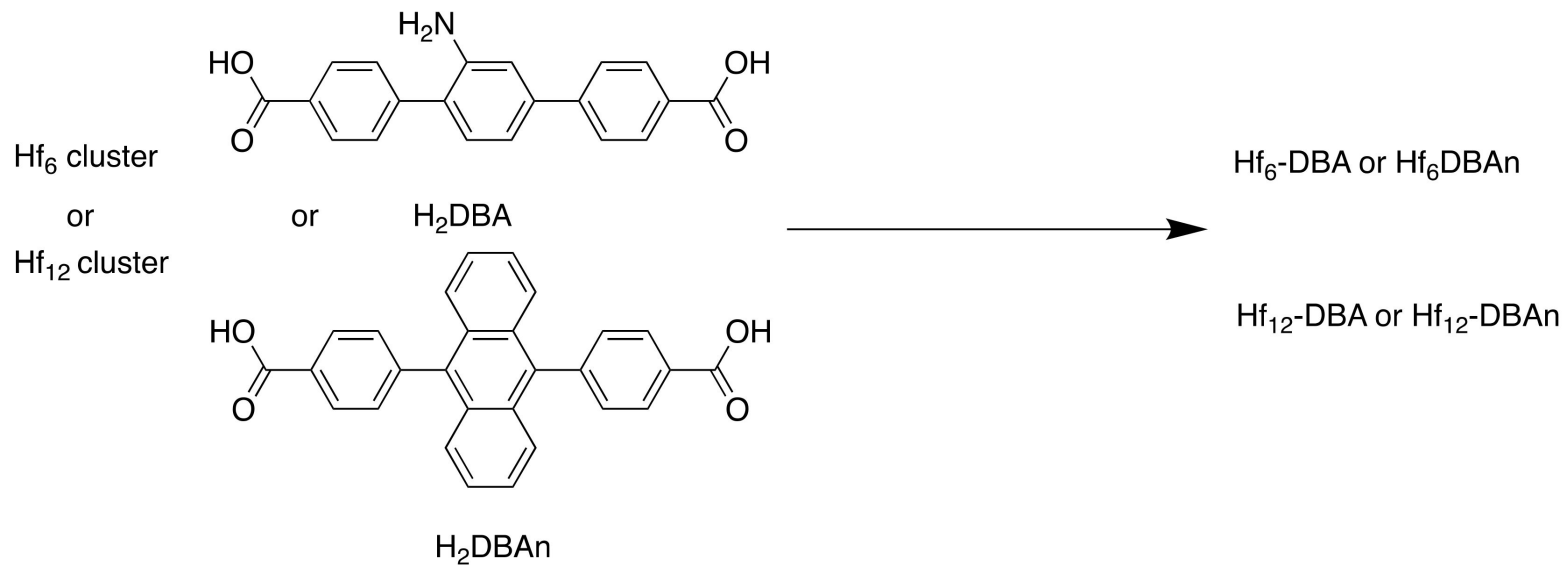
Synthesis of Hf_6_-DBA (DMF, 60 °C, 72 h), Hf_6_-DBAn (DMF, trifluoroacetic acid, 60 °C, 72 h), Hf_12_-DBA (DMF, 80 °C, 72 h) and Hf_12_-DBAn (DMF, acetic acid, water, 80 °C, 72 h).

The MOFs, Hf_6_-DBA (Hf_6_(μ_3_-O)_4_(DBA)_6_) and Hf_12_-DBA (Hf_12_(μ_3_-O)_8_(μ_3_-OH)_8_(μ_2_-O)_6_(DBA)_9_) (Figure 5), were synthesised using a solvothermal reaction (Scheme 3). The electron-dense Hf_6_O_4_(OH)_4_ and Hf_12_O_8_(OH)_14_ SBUs were chosen as X-ray absorbers that would generate ROS and allow diffusion from the channels.

Hydroxyl radical generation studies demonstrated that Hf_6_-DBA and Hf_12_-DBA both were more efficient in radioenhancing than HfO_2_ NPs. They were able to combine the nanoMOF-mediated RT with the checkpoint blockade therapy and thereby increase the RT effects from local to distant tumours.

As seen in previous examples, the unique properties of the MOF made this a successful experiment. Unlike MOFs designed for gas storage, maximised surface area does not always represent the ideal medicinal MOF. N_2_ sorption measurements were used to determine the Brunauer–Emmett–Teller (BET) surface area of the MOFs; Hf_6_-DBA was 804 m^2^ g^-1^ while Hf_12_-DBA was 464 m^2^ g^-1^. The surface area of the Hf MOFs are low in comparison to MOF-210, known to have an Langmuir surface area of over 10,000 m^2^ g^-1^ and a BET surface area of 6240 m^2^·g^−1^ [7], but for use as a radioenhancer, this parameter held less importance. Due to strong coordination between Hf^4+^ ions and carboxylate groups, both nanoMOFs were stable in aqueous suspensions, the cell culture medium and upon X-ray irradiation. The electron-dense SBUs of Hf_6_O_4_(OH)_4_ and Hf_12_O_8_(OH)_14_ absorbed X-rays to generate ROS, which cause oxidative stress to cells. The thin nanoplate structure of Hf_12_-DBA led to higher X-ray absorption and improved ROS diffusion compared to Hf_6_-DBA, highlighting the importance of considering structural effects in addition to electronic effects. This team were also behind the development of RiMO-301, a MOF which entered Phase I clinical trials in 2018 and have commented that they hope to advance further MOF-based therapies to clinical trials soon [20,49].

#### 2.1.4. MOFs as Cancer Treatments: Carbon Monoxide Therapy and Magnetic Nuclear Resonance Imaging Through MOF Modification

Further use of MOFs in medicine involves carbon monoxide (CO), a gas which, due to its toxicity, has the potential to be used in combination with chemotherapy as a cancer treatment [50]. This was first achieved in 2013 when Otterbein et al. reported that the use of CO targeted mitochondrial activity and metabolism in cancer cells resulting in the inhibition of tumour growth [51]. However, there is a need for a method of controlled administration and intracellular release of CO to minimise the risk of its toxic effects being felt by the lungs rather than the desired tumour. Previous work on precise intracellular delivery of CO has utilised metal carbonyl-caged graphene oxide (a nanomedicine), or iron carbonyl derived Prussian blue NPs [52,53]. The porous nature and tuneable structures of MOFs make them well suited to this therapy, as was recently demonstrated by Shen and co-workers [54].

**Figure 6:**
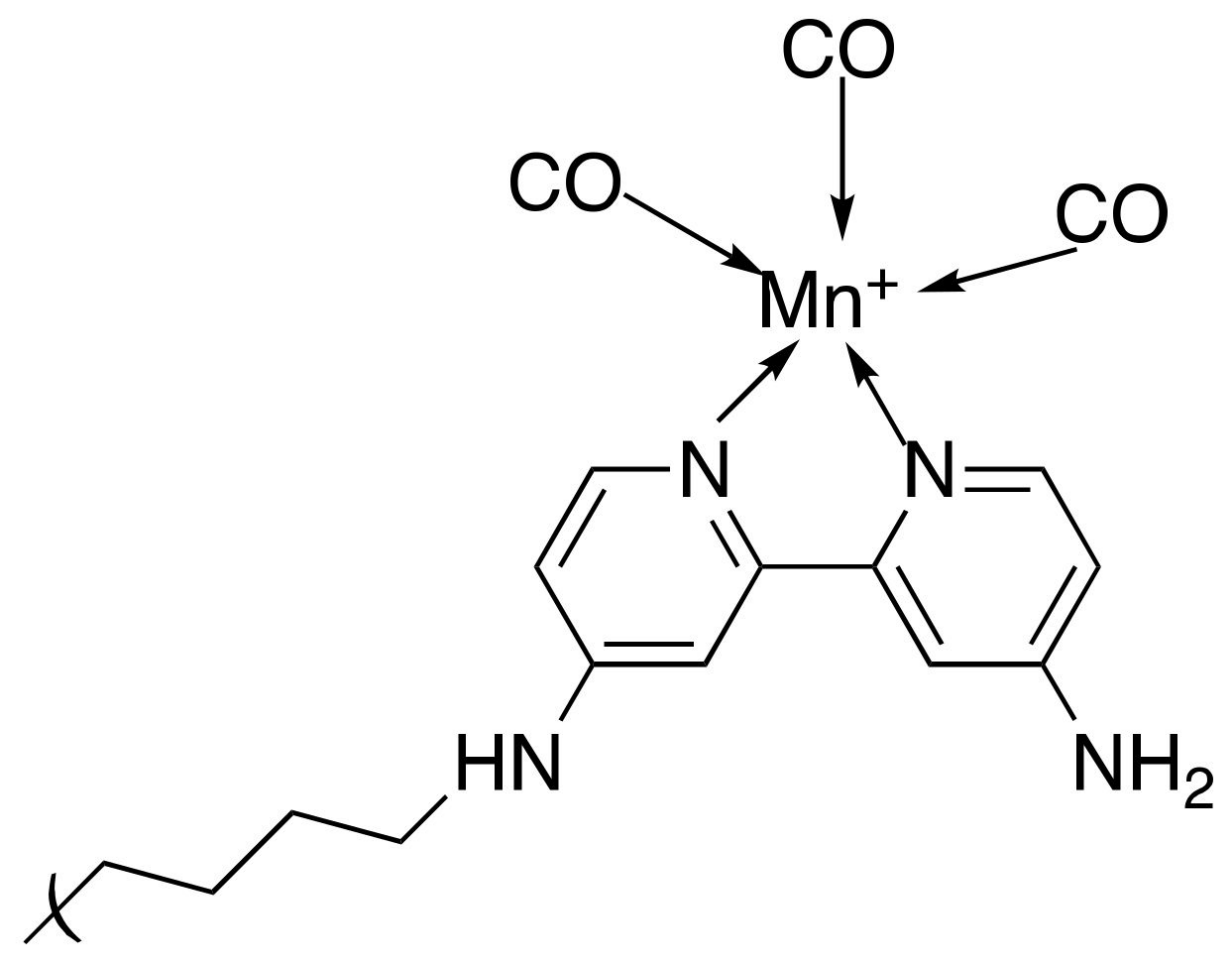
A representation of the DABPY/PEG coating on the MOF surface chelating to Mn carbonyl, the CO source.

**Scheme 4:**
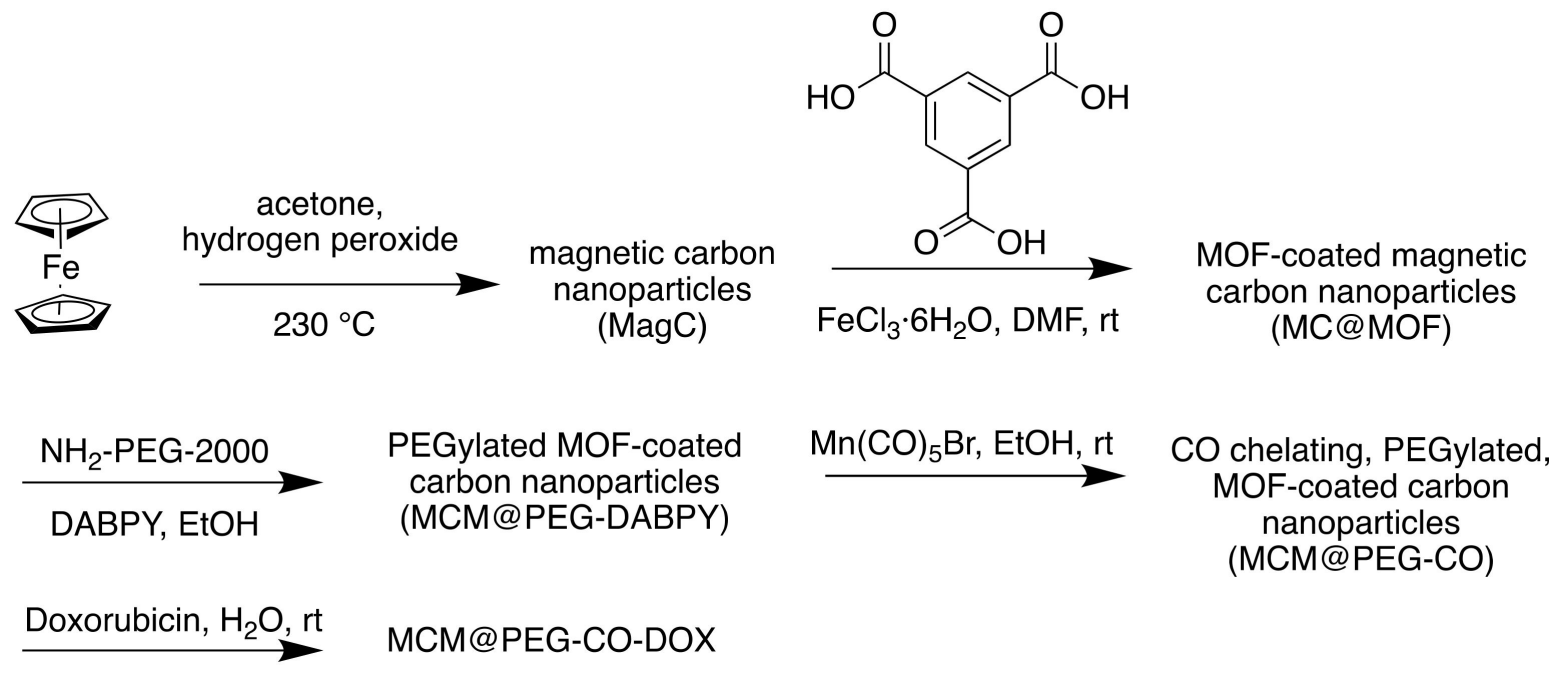
Synthesis of MCM@PEG-CO-DOX for near-infrared light-responded CO-DOX combination therapy and tumour imaging.

The chosen MOF, MIL-100 (Fe), had multiple purposes. Its pores could encapsulate the chosen chemotherapy agent, Doxorubicin (DOX) and transport it to the site of action. Additionally, the magnetic carbon nanoparticles that were coated in the MOF enabled tumour imaging such as magnetic resonance imaging (MRI) to be conducted. The MOF-coated NPs were then modified with NH_2_-PEG-2000 to increase dispersion and with 4,4′-diamino-2,2′-bipyridine (DABPY), designed to chelate to Mn(CO)_5_ as the source of CO (Figure 6). This was dispersed within Mn(CO)_5_Br/EtOH solution to obtain the CO and form MCM@PEG-CO-DOX, the therapeutic agent (Scheme 4).

Using gas chromatography-mass spectrometry (GC-MS) to determine the release ability of CO from the MCM@PEG-CO-DOX NPs, they found that irradiation with an 808 nm laser for 30 min led to release of 94.8% of the CO. Control experiments found that heat was the driving force in the release, rather than the irradiation, and additionally that this temperature rise did not affect the cell viability. This clarified that the beneficial results were due to the impact of the MOF and its cargo, rather than the conditions required for release.

Clear differentiation between the MCM@PEG-DOX NPs (without CO) and MCM@PEG-CO-DOX NPs was demonstrated, with cell survival rates of 54.9% and 38% respectively, illustrating that MOF-released CO could enhance the chemotherapeutic effect of DOX. Quantitative analysis of the T_2_ signal at the tumour site from the MRI was possible, in addition to measurement of the increase in the photoacoustic imaging intensity, demonstrating that the MCM@PEG-CO-DOX NPs were suitable for use as an imaging agent. This illustrates the benefits of using MOFs in medicine; the large pore volume (its Langmuir surface area is estimated to be >2800 m^2^ g^−1^) [55] allowed for encapsulation of the chemotherapy drug, while the use of iron(II) (with ferrocene as the source) as a component resulted in the desired magnetic properties. Further modification in the form of a PEG and DABPY coating further demonstrates the versatility of these materials.

### 2.2. MOFs as Detoxifying Agents: Adsorption and Removal of Drugs

MOFs are not solely used to bring drugs into the body—they can remove them as well. A recent paper highlighted the possibility of using MOFs as detoxifying agents [56]. Conventional treatment methods for overdose or intoxication include antidotes, gastric lavage or the use of porous media such as activated charcoal [57,58,59,60]. However, the efficacy of these methods is limited; antidotes are specific to certain toxins, and gastric lavage and charcoal remove unabsorbed drug, therefore requiring fast administration. Nanomaterials have been posited as an alternative option, utilising ligands or micelles to encapsulate or adsorb toxins in the bloodstream [60]. The use of MOFs represents a further advance in this field. High porosity and simple synthesis are valuable in a detoxifying agent, but biocompatibility and high chemical stability in the differing pH zones of the body are additional requirements for use in humans.

**Figure 7:**
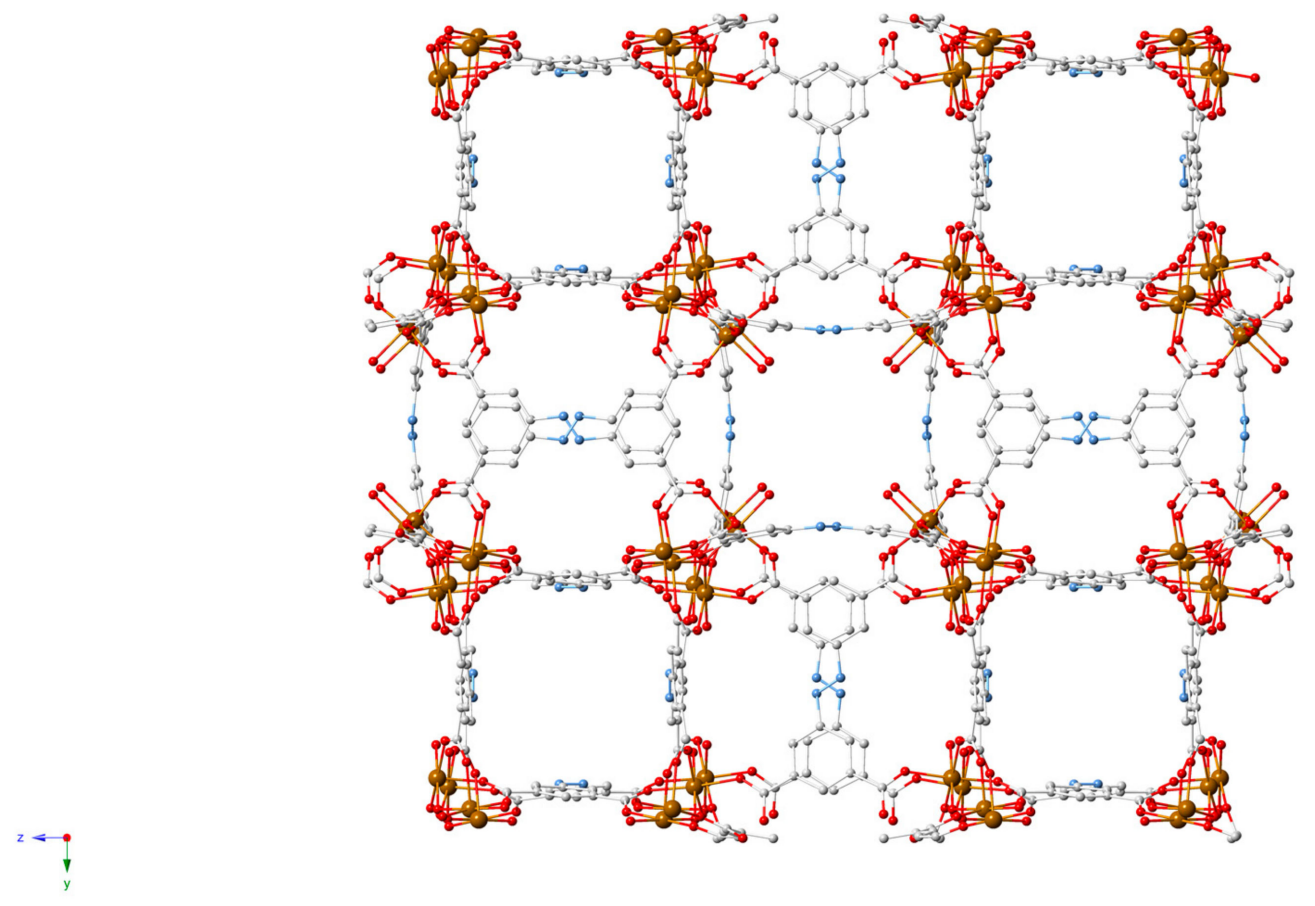
MIL-127 (Fe) crystal structure [56].

MIL-127, or Fe_3_(OH)_0.66_Cl_0.33_(C_16_N_2_O_8_H_6_)_1.5_(H_2_O)_3_·nH_2_O, was first reported by Eddaoudi et al. in 2007, using indium(III) rather than iron [61]. This MOF is both water stable and biocompatible, and has found other medicinal uses since its synthesis, such as the delivery of NO gas, reported by Serre et al. in 2014 [62]. More recently, Horcajada et al. repurposed MIL-127 as a detoxifying agent, focusing on aspirin (Figure 7) [56]. Aspirin is hydrolysed in the body to form salicylic acid, the active ingredient. Pain medication is frequently implicated in adult poison exposures, and antidotes and treatments are limited [63]. Therefore, there is an apparent demand for additional detoxifying agents and the porous nature of MOFs makes them ideally suited to this purpose.

The MOF was synthesised solvothermally using a metallic Parr bomb. As discussed in Section 2.1.1. (Structure-activity relationship between MOFs and their cargo: the effect of hydrophilicity/hydrophobicity and pore size), MIL-127 contains both hydrophilic and hydrophobic pores, has a microporous structure, and is based on iron octahedral trimers and 3,3′,5,5′-azobenzenetetracarbozylate anions [64]. MIL-127 is reported to have high porosity, with a BET surface area of 1400 m^2^ g^−1^ and pore volume of 0.7 cm^3^ g^−1^. This is an essential parameter in the case of adsorption of guest molecules.

Structural stability is required to ensure biocompatibility. This was confirmed after incubation in gastric (2 h) and intestinal (24 h) media containing aspirin. Regardless of the aspirin: MIL-127 ratio, < 9% MOF degradation was seen. Additionally, biodistribution studies demonstrated that, due to the large particle size and high chemical and structural stability, MIL-127 was not adsorbed in the intestines.

Experiments were conducted under simulated gastrointestinal (GI) conditions to determine an appropriate dose of MIL-127 in the case of an aspirin overdose. At a 1.5:1 ratio of aspirin: MOF, 39.4% of the salicylates were adsorbed, compared to 25.2% at a 3:1 ratio. Despite the (expected) trend between higher MOF content and higher amount of removed aspirin, the drug loading (g drug per g MOF) did not depend on the amount of MOF. The maximum aspirin capacity reached was around 0.14 g g^−1^ in both cases. Importantly, after exchange from gastric medium to intestinal medium, there was no detected release of salicylates, which indicated both gastric stability and high affinity of the drug to the MOF. Therefore, while in terms of adsorption, MIL-127 showed lower levels than activated carbon, there was a definite advantage seen in the GI tract, as MIL-127 was able to retain the aspirin.

When administered orally in rats, after 24 h, the plasma and urine concentrations of salicylates were reduced threefold in the presence of MIL-127, reaching similar results as previously published for the adsorption of aspirin by activated carbon [65]. This work illustrates the diverse range of biomedical applications that make use of the porous nature of MOFs.

### 2.3. MOFs as Catalysts for the Degradation of Pharmaceutical Contaminants in Wastewater

**Figure 8:**
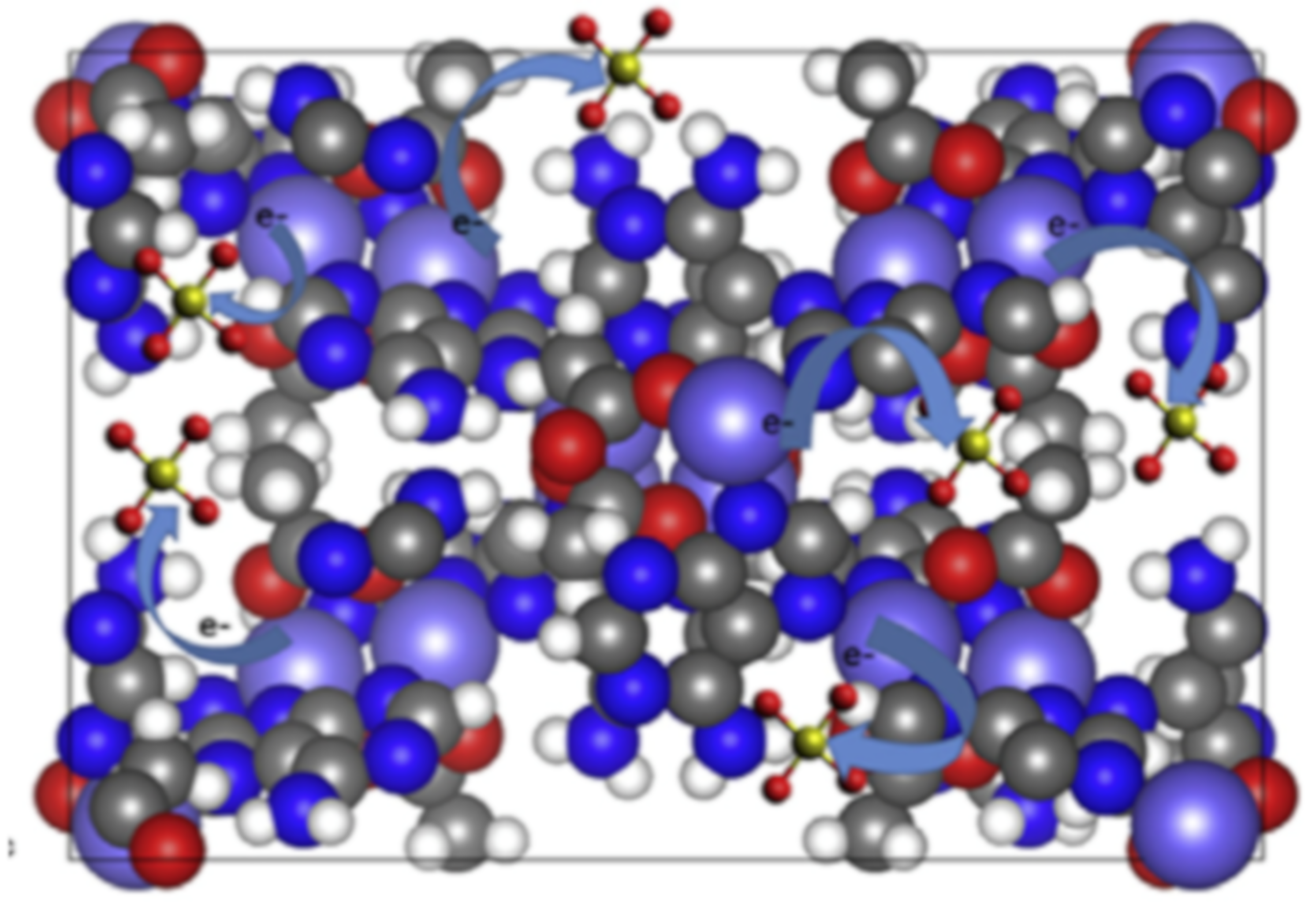
Representation of activation of peroxymonosulfate (PMS), used in catalytic degradation processes, by bio-MOF-11 where cobalt is purple, carbon is grey, oxygen is red, hydrogen is white and nitrogen is blue [68]. Reprinted from Chemosphere, 196, M. R. Azhar, P. Vijay, M. O. Tadé, H. Sun and S. Wang, Submicron sized water-stable metal organic framework (bio-MOF-11) for catalytic degradation of pharmaceuticals and personal care products, 105-114, Copyright (2019), with permission from Elsevier.

MOFs are valuable catalysts, including in medicinal contexts. Sulfonamide antibiotics, such as sulfachloropyradazine (SCP), have toxic effects on marine life, yet overuse has resulted in frequent detection of SCP in wastewater [66]. Parabens (esters of para-hydroxybenzoic acid (*p*-HBA) are found in pharmaceuticals, glues, animal feeds, personal care products and other substances [67]. These degrade into various derivatives, including *p*-HBA, which has been shown to have adverse health effects in men and women. These adverse effects demand the removal of these water contaminants. Activated carbon and zeolites have been used in the removal of parabens and their derivatives, but issues including recycling difficulties and low adsorption levels remain [68]. Therefore, water-stable MOFs are essential materials to control levels of water contaminants via adsorption and catalytic reactions. The development of a highly water stable, biocompatible, cobalt and adenine based MOF, bio-MOF-11-Co, facilitated the degradation of parabens, via activation of the oxidising agent peroxymonosulfate (PMS) (Figure 8) [68].

**Scheme 5:**
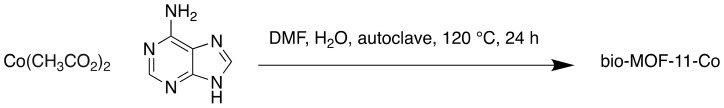
Synthesis of bio-MOF-11-Co.

Bio-MOF-11-Co was synthesised from cobalt acetate and adenine, an amino acid (Scheme 5) [69]. Water stability was a crucial property in this study. Previous studies have reported that copper is active in catalytic reactions in wastewater treatment processes; therefore, two other MOFs, copper benzene tricarboxylate (CuBTC) and bio-MOF-11-Cu, were tested. However, degradation in water was observed for both materials, marking them unsuitable for wastewater purification. Bio-MOF-11-Co was stable in water and efficiently activated PMS to enable degradation of organic molecules, and the metal leaching observed was negligible. Furthermore, the catalyst was reused three times with only water washing and drying at 130 °C in between. Cobalt, a constituent of the vitamin B_12_, is essential for life in minute amounts [70]. However, in larger amounts cobalt can be poisonous, with an ‘immediate danger to life and health’ (IDLH) value of 20 mg m^-3^ [71], making the true biocompatibility of this MOF questionable.

**Figure 9:**
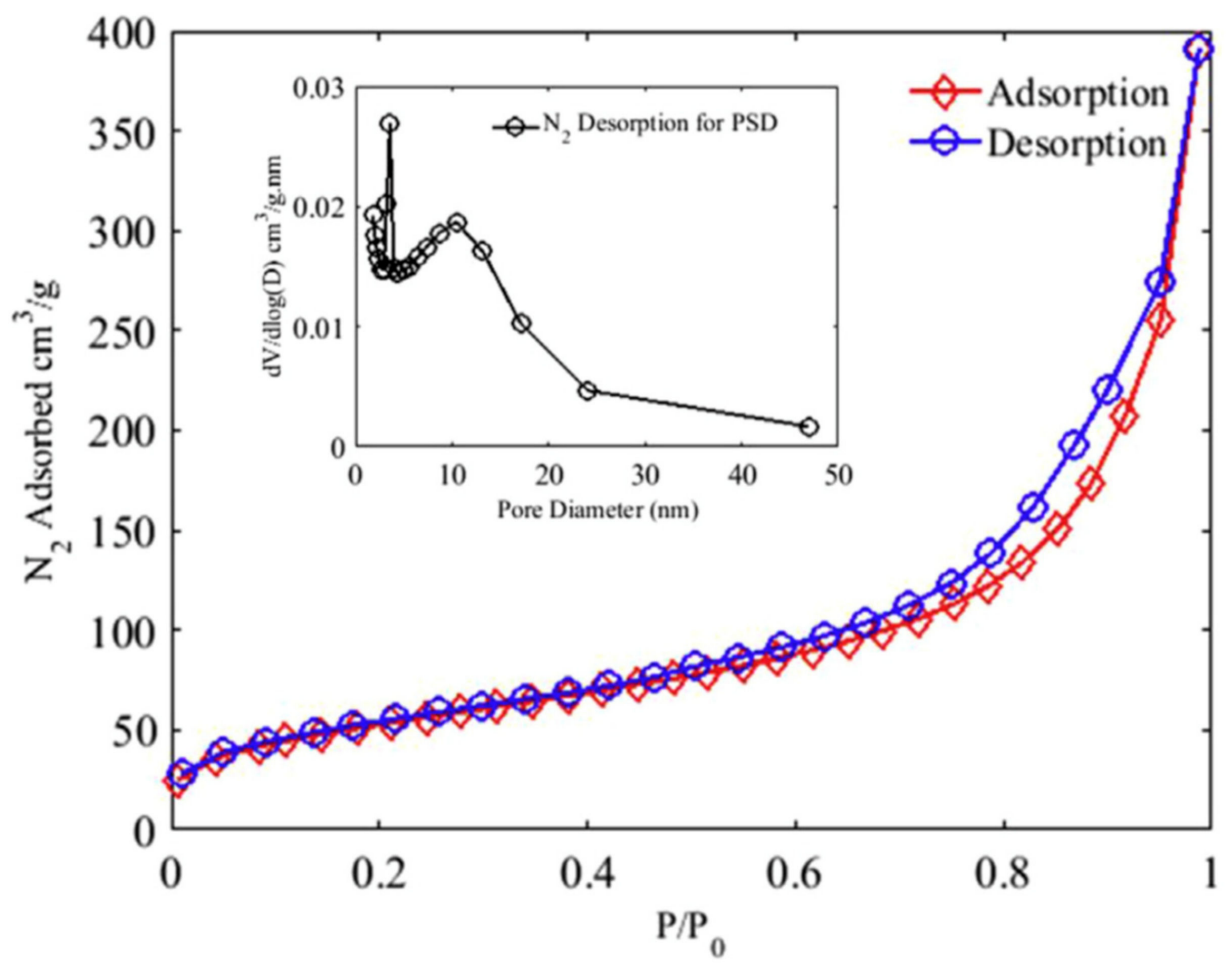
N_2_ adsorption-desorption isotherms of bio-MOF-11-Co used to calculate surface area and pore size distribution (PSD) using the Barrett-Joyner-Halenda method [68]^.^ Reprinted from Chemosphere, 196, M. R. Azhar, P. Vijay, M. O. Tadé, H. Sun and S. Wang, Submicron sized water-stable metal organic framework (bio-MOF-11) for catalytic degradation of pharmaceuticals and personal care products, 105-114, Copyright (2019), with permission from Elsevier.

Significant attention was focused on confirming the significance of the Lewis basic sites of the adenine ligands within the MOF. Previous studies have highlighted the effect of strongly basic N atoms in catalytic processes [72]. This, combined with the variable oxidation states of cobalt, allowed the catalytic degradation to occur. It was proposed that the sulfate and hydroxyl radicals were generated by electron transfer. Bio-MOF-11-Co was shown to be mesoporous via a characteristic type IV nitrogen adsorption-desorption isotherm (Figure 9).

The larger size of mesopores allows less resistance when guest molecules enter the pores, resulting in better adsorption [73]. The researchers noted that in terms of catalyst and PMS loading and reaction time, bio-MOF-11-Co was superior to other catalysts for the degradation of *p*-HBA and SCP. This could be attributed to the aforementioned range of properties of this MOF that make it well suited to the task of catalytic degradation.

## 3. Bioinspired MOFs

While there are numerous examples of MOFs being used as drug delivery systems via encapsulation of an API, less prevalent is the use of biologically relevant molecules as linkers. Taking inspiration from biological processes by using biocompatible metals and materials can reduce the risk of toxicity related to release of MOF components once degraded [21]. However, the synthesis of permanently porous and stable MOFs from biomolecules is challenging, in part due to the flexibility of large molecules with high numbers of sp^3^ carbons, and a limited number of coordination sites.

### 3.1. APIs as Linkers

Using APIs which have been rigorously tested in clinical trials ensures the maximum dosage and related toxicity is known. Additionally, the prevalence of aromatic motifs in APIs can provide more rigid structures. Incorporation into a MOF provides a novel formulation for the API, potentially improving dosage or solubility issues. This has been exemplified in works by Serre, combining nicotinic acid and iron (2010) [74], and Burrows and Spencer, using zinc or bismuth coordination networks (CN) involving deferiprone, an iron overload drug, as a chelating ligand [75,76]. The latter work observed drug release from the zinc-CN under acidic conditions, while phosphate buffered saline (PBS) released deferiprone from the bismuth analogue, and in Serre’s study degraded bioMIL-1 to release nicotinic acid.

#### 3.1.1. APIs as Linkers: Olsalazine and M_2_(olz)

**Figure 10:**
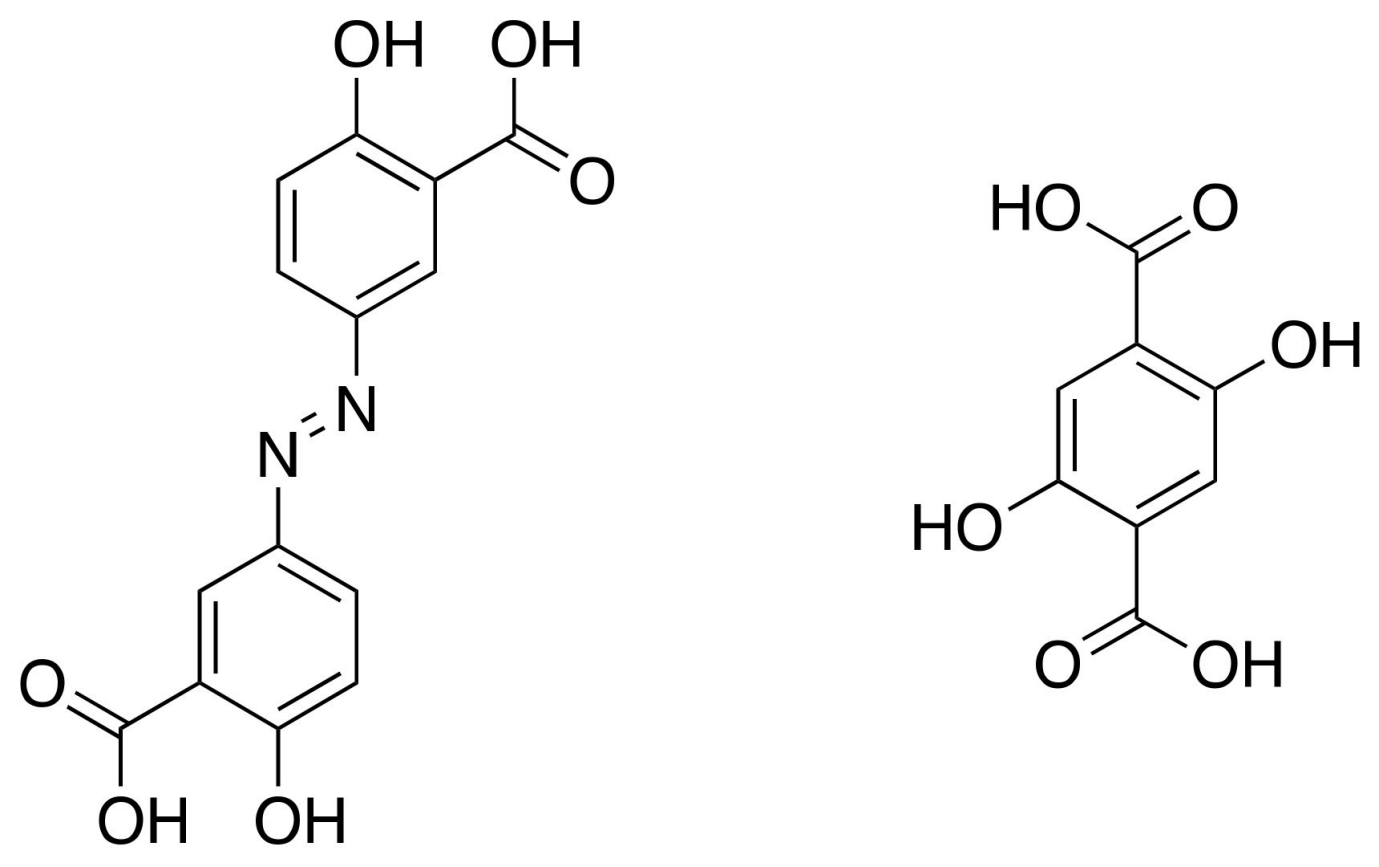
Olsalazine (left) and dobdc.

Olsalazine, a prodrug of the anti-inflammatory 5-aminosalicylic acid, is widely used in the treatment of gastrointestinal diseases such as ulcerative colitis [77]. Additionally, olsalazine has been shown to inhibit the development of colorectal cancer [78]. Due to the relative scarcity of MOFs constructed wholly from biorelevant materials, without requiring ancillary ligands as in previous olsalazine structures [79,80], in 2016 Long et al. sought to expand a series of isoreticular MOFs of the series M_2_(dobdc) (dobdc = 2,5-dioxido-1,4-benzene-dicarboxylate) to create an olsalazine-based MOF with pores of sufficient size to encapsulate additional APIs [81]. M_2_(dobdc) structures consist of dicationic metals and dobdc^4-^, a tetraanionic linker, and have 1D hexagonal pores. Olsalazine has the same coordinating functionalities as the dobpdc^4-^ ligand, but is slightly longer, which the authors theorised could result in expanded pore sizes (Figure 10).

**Scheme 6:**
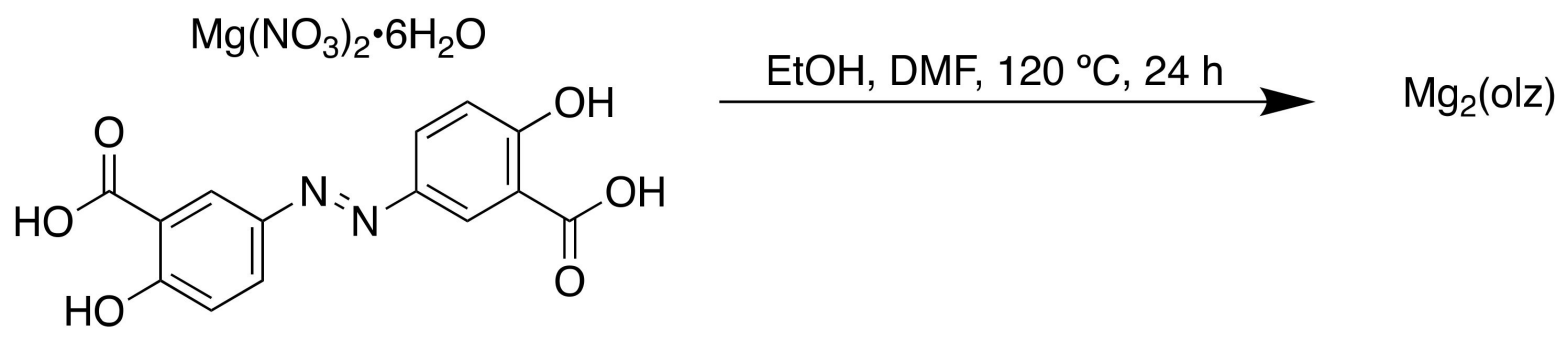
Synthesis of Mg_2_(olz).

The MOFs were synthesised using solvothermal techniques (Scheme 6), resulting in isostructural frameworks with mesopores of approximately 2.7 nm and 86 *wt* % API.

As illustrated in Table 2, the surface area of the MOF varied with each metal, with Mg having the largest BET and Langmuir surface area.

In addition to the therapeutic properties of olsalazine, the large pores of Mg_2_(olz) allowed the encapsulation of a second therapeutic agent, exemplified here with the model drug phenethylamine (PEA) to form Mg_2_(olz)(PEA)_2_. Degradation of the Mg_2_(olz)/Mg_2_(olz)(PEA)_2_ framework to release olsalazine/olsalazine and PEA was observed under physiological conditions, reinforcing the suitability of this structure as a bioactive agent and demonstrating that the MOF could be used as a co-delivery agent.

The use of DMF and methanol in the synthesis of Mg_2_(olz) highlights a pertinent question in the field of bioMOFs, as to whether a material can be described as biocompatible if there is the possibility of toxic solvents remaining within the MOF framework. It was reported that Mg_2_(olz) was heated under argon and vacuum to remove the residual or coordinated solvent, but it could be alleged a risk remains, and use of biocompatible solvents should be prioritised. No in vivo studies were performed to investigate whether olsalazine in MOF form resulted in improved bioavailability. However, the formulation of olsalazine into a MOF has advantages over free olsalazine as it offers the potential to deliver multiple APIs within one unit.

#### 3.1.2. APIs as Linkers: Curcumin and Medi-MOF-1

**Figure 11:**
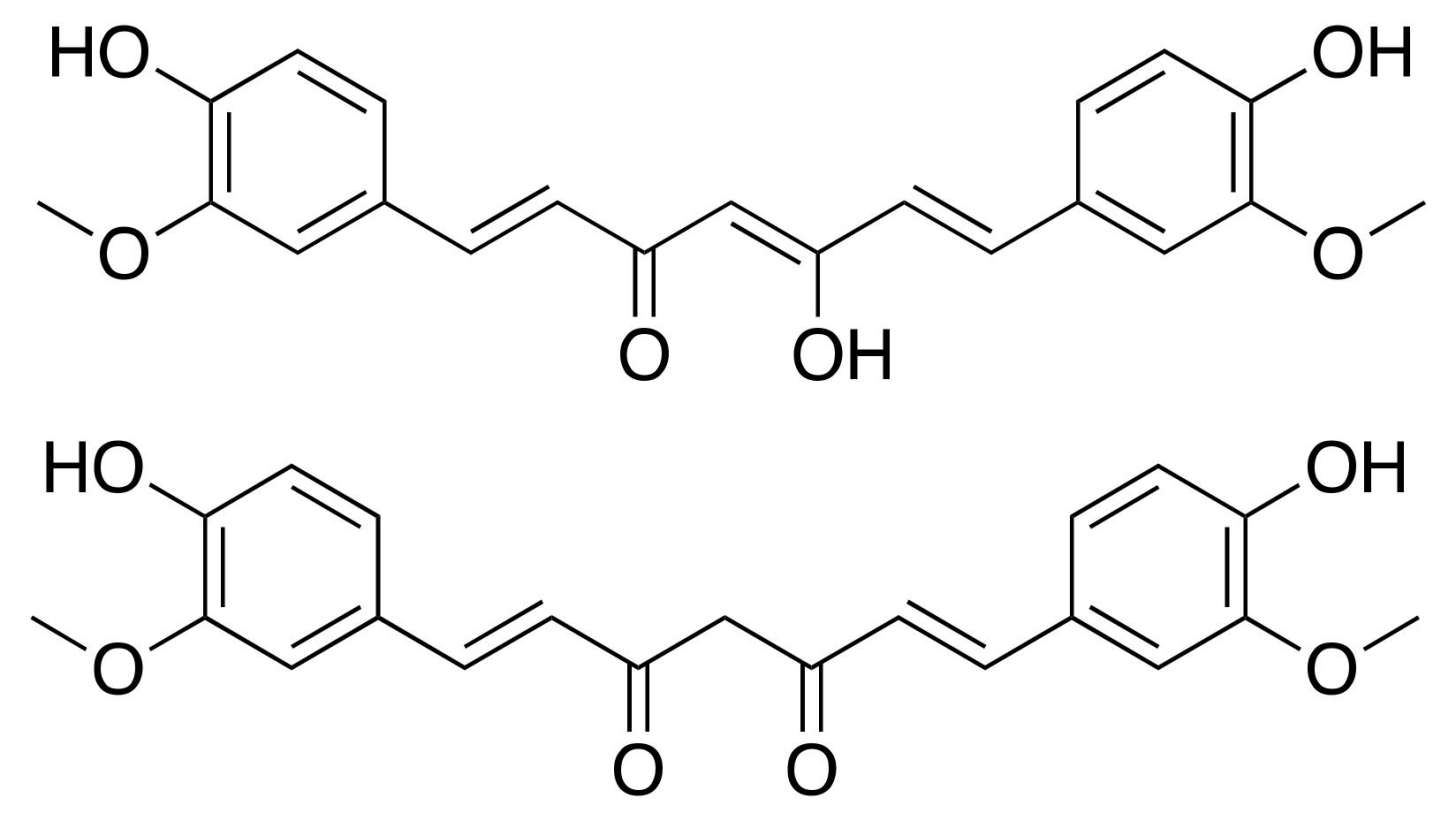
Curcumin in enol (top) and ketone (down) form.

A further example utilises the bioactive pharmaceutical ingredient curcumin (Figure 11) [82]. Curcumin has been shown to possess anti-oxidative, anti-inflammatory and anticancer activity [83], and is safe for humans even at high oral doses of 12 g per day [84]. This biocompatibility enables curcumin to be used as a non-toxic building block in MOFs designed for biomedical applications.

**Scheme 7:**
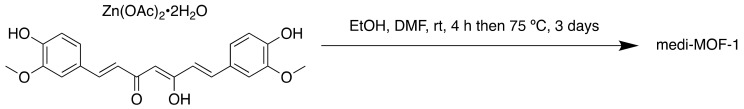
Synthesis of medi-MOF-1.

The MOF was synthesised solvothermally using the biocompatible metal Zn with curcumin in the enol form (Scheme 7) [85,86]. Medi-MOF-1 is formed of SBUs consisting of Zn(II) metal-oxo trimers, bridged by hydroxyl O to six other trimers. The O of the 1,3-diketone moiety in its enol form acts as the third coordination site of the linker. Zn-curcumin chains are formed into a 3D distorted primitive cubic (pcu) net by Zn covalently bonding to O atoms of the 1,3-diketone group on other chains. Pores of ~11Å are located in the centre of the pcu lattice cell, with open channels of 6–8 Å. along with specific directions.

Previous studies have demonstrated the ability of curcumin to inhibit the growth of pancreatic cancer cells [87,88]. An in vitro cytotoxicity assay assessed the differences between zinc acetate dehydrate (Zn(OAc)_2_•2H_2_O), curcumin and medi-MOF-1, finding similar cytotoxic efficacy between medi-MOF-1 and curcumin. The authors tested the MOF as a co-delivery system using the model drug ibuprofen, resulting in drug loading of about 0.24 g g^−1^. Under physiological conditions (PBS at 37 °C), 97% of the encapsulated ibuprofen was released within 80 h. This occurred in two distinct stages, with over 50% of the drug, which had formed weaker interactions, released within two hours, and the remaining encapsulated drug, which had made stronger interactions with the MOF framework, slowly releasing in the remaining period. Medi-MOF-1 was unstable in PBS and degraded in the in vitro buffer conditions with 30% of the linker released within the first hour. The authors noted that the synergistic release of curcumin and the loaded drug means medi-MOF-1 acts strictly as a co-delivery system. Medi-MOF-1 exemplifies the potential for MOFs with bioactive linkers to be used in medicine, as they can act as a dual therapy similar to the MOF used for radiotherapy discussed in 2.3.1.

#### 3.1.3. APIs as Linkers: Nalidixic Acid and Mg-MOF

More recently, Mg- and Mn-based MOFs were synthesised using the antibiotic agent nalidixic acid [89]. Nalidixic acid is an antibiotic with high bactericidal and bacteriostatic activity against Gram-negative bacteria, yet its low aqueous solubility leads to poor oral bioavailability [90,91]. Nalidixic acid is particularly relevant to MOF formulation as a synergetic effect of a metal ion to quinolone drugs has been observed, prompting several metal-based nalidixic acid complexes to be synthesised in the past few years [92].

**Scheme 8:**
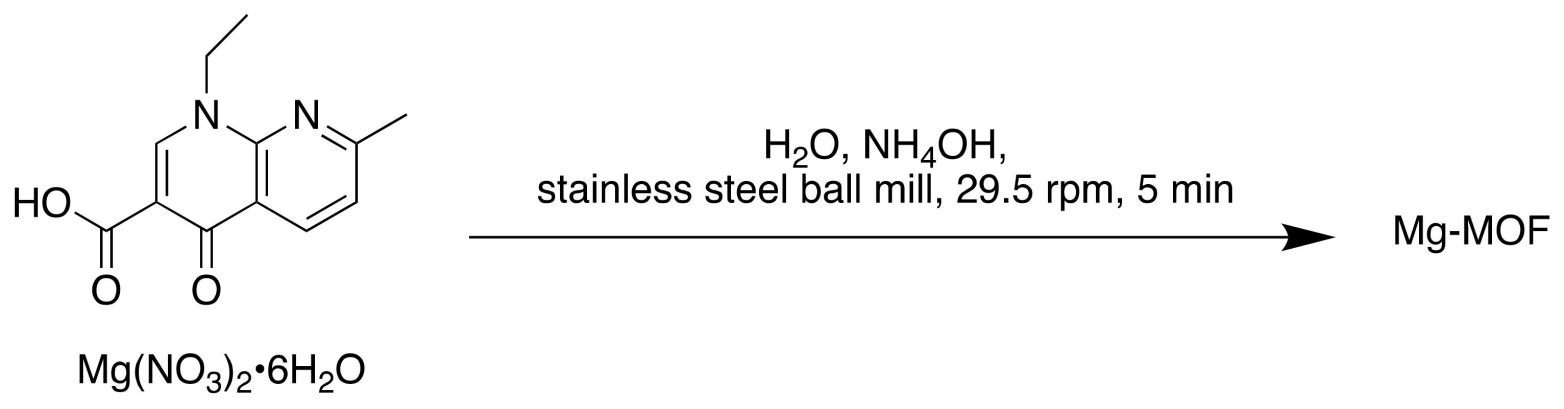
Synthesis of Mg-MOF.

Using mechanochemistry, two bioMOFs based on nalidixic acid and the biocompatible metals Mn and Mg were synthesised (Scheme 8). Water and ethanol were used as solvents during crystal growth by slow evaporation, ensuring full biocompatibility in case of residual solvent in the MOF.

Importantly, the incorporation of nalidixic acid into a MOF enhanced the solubility of the materials in aqueous media. This was tested using high performance liquid chromatography (HPLC) and was attributed to the carboxylate character of the MOFs. While both MOFs were tested on Gram-positive bacteria (Staphylococcus aureus and Enterococcus faecalis), Gram-negative bacteria (*Pseudomonas aeruginosa* and *Escherichia coli*), and yeast strains (*Candida albicans* and *Saccharomyces cerevisiae*), the most significant inhibition of growth was observed against the Gram-negative bacteria E. coli. Mg-MOF demonstrated a bactericidal effect against the Gram-negative bacteria, while Mn-MOF induced a bacteriostatic effect. The antimicrobial activity of both bioMOFs was higher than the nalidixic acid activity alone, demonstrating that an increase in antibiotic efficiency may be achieved by delivery in the form of bioMOFs.

There is clear potential for further bioapplications using biocompatible metals and linkers. MOFs have demonstrated potential as drug delivery agents and use of biocompatible or API linkers can limit potential toxicity issues from the degradation of a MOF within the body. Additionally, co-delivery systems or dual therapeutics using an encapsulated agent in combination with the linker can reduce the number of doses of various APIs, potentially improving patient compliance.

Issues remain, including lack of metal-coordinating functional groups on APIs limiting the ability to form 3D structures, necessitating ancillary linkers. Additionally, harmful solvents, such as DMF, are frequently used in MOF synthesis and could have a toxic effect on the body unless entirely removed from the structure [93,94]. As stated previously, the flexibility of organic ligands can lead to instability in MOFs. However, the number of sp^3^ carbons in a drug has been correlated to improved solubility and likelihood of clinical success [95], and so use of APIs in MOF synthesis may remain challenging. 

### 3.2. Amino Acids and Endogenous Small Molecules as Linkers

In addition to using APIs as MOF constituents, amino acids have proved useful in the synthesis of medicinally relevant MOFs. These biologically essential compounds are used in the body for the synthesis of proteins or as a source of energy via oxidation to urea and CO_2_. Amino acids (except glycine) possess inherent chirality, which has been utilised to create homochiral MOFs for applications, such as separation of chiral structures [96]. 1D and 2D CPs can form due to the single carboxylate coordinating group which can be remedied through the use of ancillary ligands and mixed systems [21].

### 3.3. The Krebs Cycle

**Figure 12:**
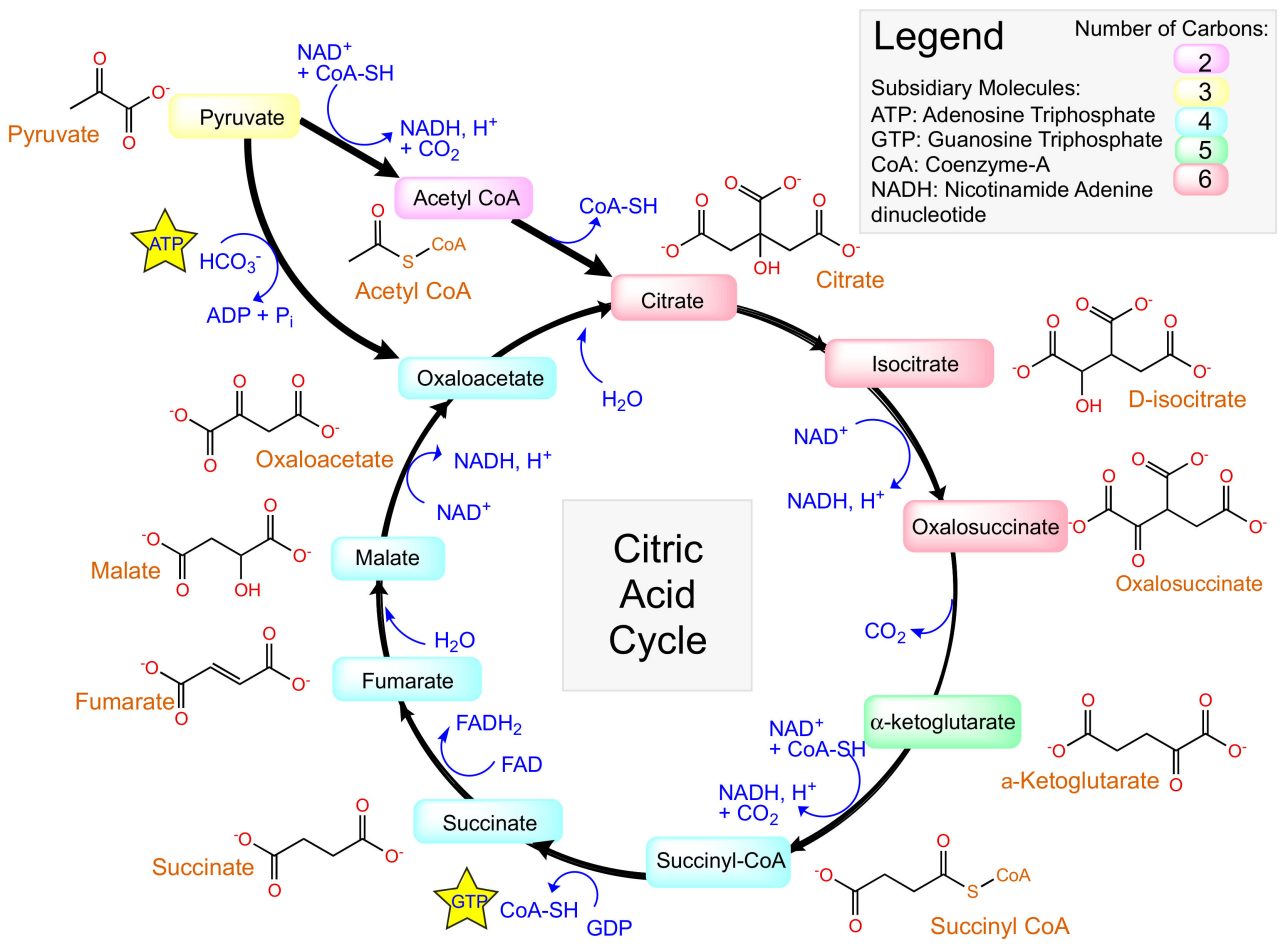
The Krebs cycle, or citric acid cycle.

The Krebs cycle, or citric acid cycle (CAC) (Figure 12), is a process within the body to release energy through the oxidation of acetyl-coenzyme A (CoA) to adenosine triphosphate (ATP) [97]. Acetyl-CoA is derived from carbohydrates, fats and proteins. Precursors of several amino acids are made during the cycle in addition to the reducing agent nicotinamide adenine dinucleotide (NADH).

Taking inspiration from biologically relevant materials such as amino acids and other small molecules involved in the Krebs cycle can ensure biocompatibility in a MOF.

#### 3.3.1. Citric Acid

The starting point of the CAC is the synthesis of citrate from acetyl-CoA. Citric acid has been used as a building block for numerous MOFs due to its three carboxylate groups, which allow trivalent coordination. The first neutral ferrous citrate MOF was reported in 2013, a 1D CP made pseudo-3D by hydrogen bonds between chains [98]. Iron readily forms complexes with oxygen, is biocompatible and is involved in several biological processes [99].

**Scheme 9:**
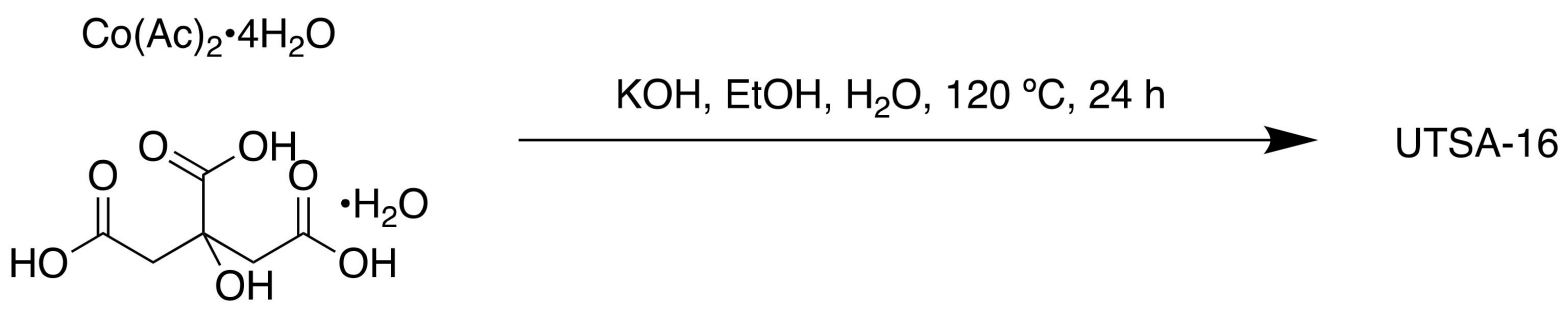
Synthesis of UTSA-16.

A more recent example uses citrate in the synthesis of a MOF designed for oxygen evolution reaction (OER) electrocatalysis [100]. The OER, 2H_2_O → O_2_ + 4H^+^ + 4e^−^, is an oxidative half-reaction of water splitting, to which much research has been directed at the development of efficient catalysts to overcome the slow kinetics and high overpotential which limit this technology [101]. Their porous nature enables MOFs to act as efficient OER electrocatalysts, with the built-in pores and open channels allowing space for the diffusion of reactants and the generated oxygen gas [102]. UTSA-16 was synthesised solvothermally (Scheme 9) and consists of tetranuclear cobalt citrate clusters in which citrate O chelate octahedral cobalt atoms to form the Co_4_O_4_ cubane motif. This mimics the natural photosystem II and is successful in OER [103].

These clusters act as octahedral linkers and tetrahedral Co(II) atoms as trigonal nodes. The MOF had high activity in alkaline medium, and the authors reported similar efficiency to the benchmark OER catalyst RuO_2._ The success of UTSA-16 highlights the value of bioinspired MOFs, not only in medical and bio-related applications, but additionally for more common uses for MOFs, such as catalysis.

#### 3.3.2. Lactic Acid

Recent studies have suggested that lactate, found in the Cori cycle, can be used as a C source in the Krebs cycle [104]. In 2017, Yaghi et al. synthesised two chiral lactic acid-based MOFs, Ca_14_(L-lactate)_20_(acetate)_8_(C_2_H_5_OH)(H_2_O) (MOF-1201) and Ca_6_(L-lactate)_3_(acetate)_9_(H_2_O) (MOF-1203) [105]. As with the use of MOFs as drug delivery systems, concerns about the effect that non-biocompatible metals and linkers may have on natural systems inspired the development of fully biocompatible MOFs. Degradation of the MOF is required to release the encapsulated pesticide, and therefore the toxicity of the metal and linker on the environment must be considered; Yaghi’s design of a biological acid-based MOF circumvented this issue. Many fumigants, a common form of pesticides, are commonly applied by drip irrigation or shank injection [106]. However, the volatility of conventional pesticides, such as 1,3-dichloropropene and chloropicrin, results in loss and air and groundwater pollution in addition to hazards to users. Porous solids including activated carbon, clay or alumina and adsorption resins have been proposed for use as sorption formulations, allowing adsorbed fumigant to be slowly released [105]. However, limited biodegradability can lead to accumulation of carrier materials, adding to the environmental impact of pesticide use.

Use of ethanol as the solvent ensured full biocompatibility, removing the risk of residual toxic solvents. Acetate was used as an additional ligand, resulting in a three-dimensional arrangement of Ca(−COO, −OH) polyhedra. These contained 1D pores with apertures and internal diameters of 7.8 and 9.6 Å (MOF-1201) and 4.6 and 5.6 Å (MOF-1203). These MOFs represented the first examples of extended porous structures based on Ca(II) and lactate, perhaps reflected by the very low permanent porosity of 430 (MOF-1201) and 160 m^2^ g^−1^ (MOF-1203). Both the high coordination number and geometries of Ca(II) and the flexibility of bio-relevant linkers can result in the formation of dense, nonporous structures, and prior to this work no Ca(II) MOF containing a naturally occurring linker had been reported [105]. MOF-1201 was chosen to be tested as a formulation for the volatile fumigant *cis*-1,3-dichloropropene due to its higher porosity.

Degradation of MOF-1201 to its constituents in water was observed, with 120 ± 10 g dissolved in 1 L of water. The saturated solution had a pH of 7.6, further evidence for its suitability as a pesticide carrier with minimal environmental impact, other than leaving calcium as a nutrient in the soil. Aqueous degradation points to applications not only as a pesticide carrier but medicinally too.

#### 3.3.3. Succinic Acid

In the Krebs cycle, succinate is oxidised to fumarate by succinate dehydrogenase. Succinic acid is a commonly used linker in MOFs, combined with metals including cobalt and terbium [107,108]. As a biocompatible material, materials made from succinic acid have the potential to be used as DDS or adsorbents with reduced environmental impact.

In 2018, a biocompatible MOF consisting of aluminium and succinic acid was reported for the removal of azo dyes in wastewater [109]. Synthetic dyes are common aqueous pollutants; azo dyes, in particular, have highly toxic effects and require complete removal from wastewater in order to mediate any ill effects on aquatic or human life [110,111]. Adsorption techniques hold several advantages over other methods such as electrochemical coagulation or ozonation, mainly in terms of simplicity, selectivity and minimal secondary pollutants [112,113]. Using MOFs as absorbents, due to their porosity, has been an area of significant interest.

**Scheme 10:**
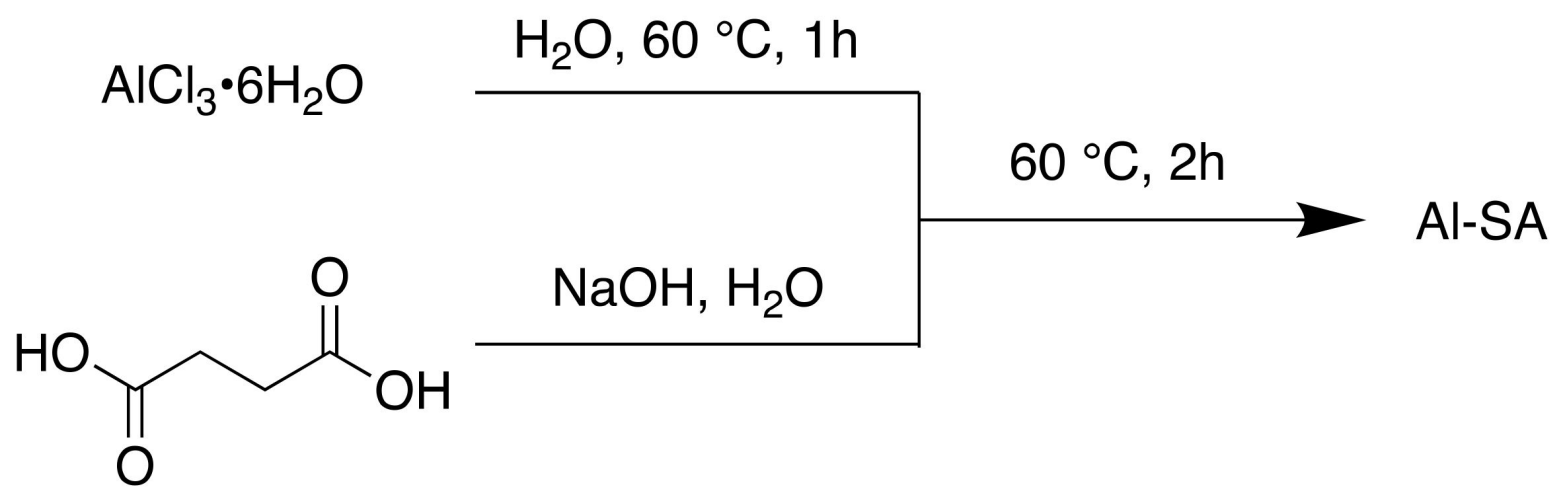
Synthesis of Al-SA MOF.

The MOF reported here, Al-SA, was synthesised using water as the sole solvent (Scheme 10).

Not only this approach is more environmentally friendly, but the risk of toxic solvents entering wastewater is also removed. Toxic solvents, such as DMA and DMF, are frequently used in the synthesis of MOFs, producing secondary pollutants [93,112].

Al-SA was reported to have a BET surface area of 358.03 m^2^ g^−1^ and pore volume of 0.399 cm^3^ g^−1^. This low BET surface area could be attributed to the flexibility of the succinic acid linker, which, as mentioned, can affect the porosity of MOFs. Al-SA is mesoporous with pores of 4.96 nm. Despite this, it had a similar or higher maximum adsorption capacity to many other adsorbents, including powder activated carbon (PAC) (Table 3, Table 4). Al-SA demonstrated high affinity towards both the synthetic azo dye AB1 and another azo dye, A07, and had a >97.7% removal rate of AB1 across all pH ranges tested.

#### 3.3.4. Fumaric Acid

Other bioinspired MOFs have been used for biological and health-related applications. Fumaric acid or fumarate is a common food additive and plays a role in the CAC [120]. Recently a fumaric acid-based MOF was developed for the removal of fluoride from brick tea [121]. Brick tea is consumed in many parts of the world, but its consumption can result in side effects [122,123]. Mature tea plants can absorb and store high levels of fluoride from the air and soil [124], which can pose a danger to regular consumers of brick tea as fluoride is released from the tea during infusion and is highly bioavailable [125]. While small amounts of fluoride are essential for health, large quantities can cause fluorosis. To mitigate this health risk, absorbents can be used to remove F^-^ from the soil.

**Scheme 11:**
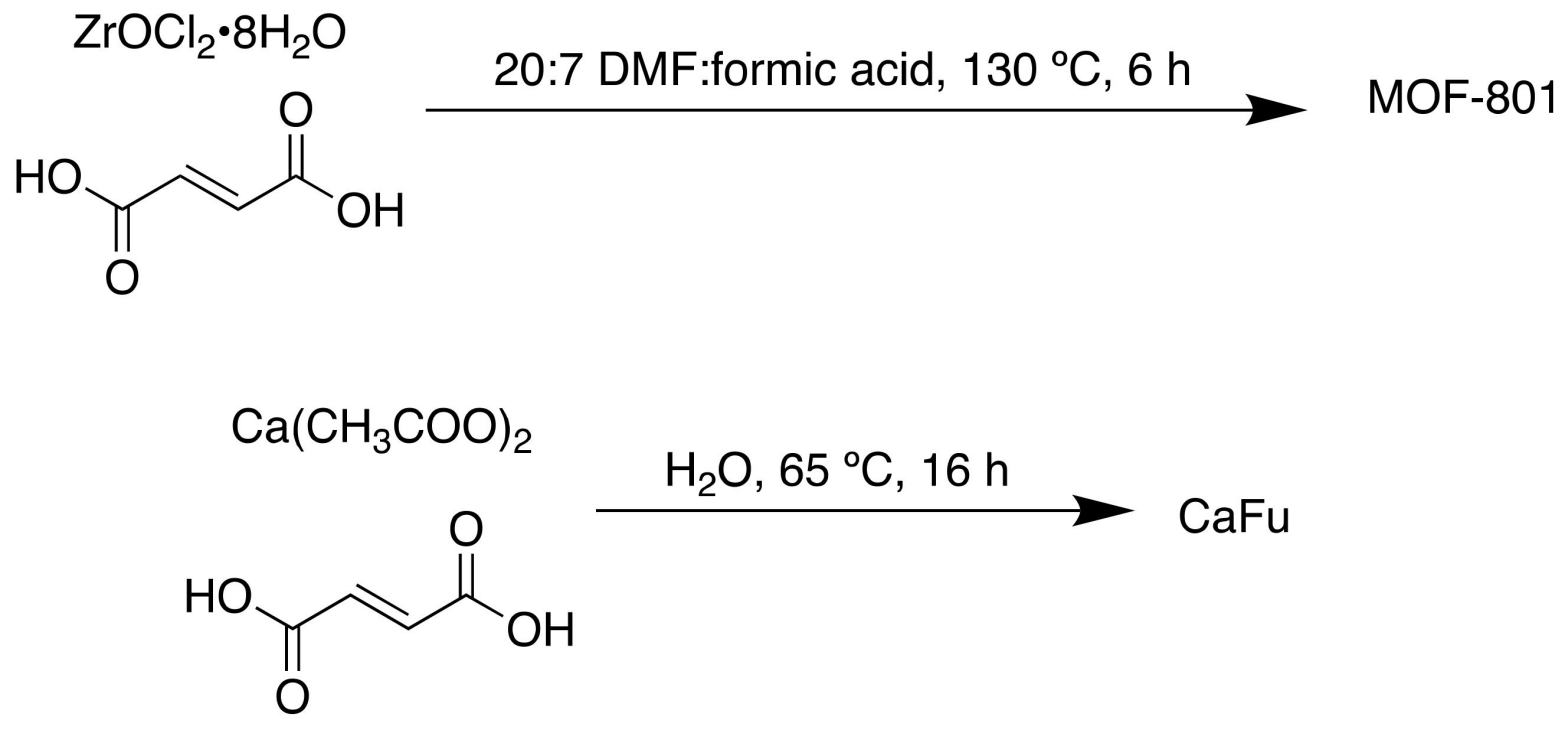
Synthesis of MOF-801 and CaFu.

Zirconium (MOF-801) and calcium (CaFu) (non-toxic) fumaric acid MOFs were synthesised solvothermally (Scheme 11) and used for this purpose.

The pH of the solution has an impact on the surface charge of the adsorbent; acidic pH resulted in MOF-801 being positively charged, facilitating fluoride adsorption. Brick tea has a pH value of 5.4 and as such experiments were carried out at this pH. Within 5 min, the non-biocompatible Zr MOF was shown to remove almost 80% of fluoride ions in the brick tea infusion. The biocompatible Ca-based MOF was less efficient, with 37% fluoride removed when using 2.0 g L^−1^ adsorbent compared to almost 70% F^−^ removal under the same conditions for MOF-801. However, for both MOFs, only minor loss of caffeine was observed within 30 min, indicating high selectivity for fluoride over other components in the brick tea infusion.

FTIR, EDX and XPS indicated that the hydroxyl groups in the MOFs were substituted by fluoride. The high selectivity and adsorption rates of CaFu indicate promising applications as an adsorbent.

#### 3.3.5. L-Malic Acid

**Figure 13:**
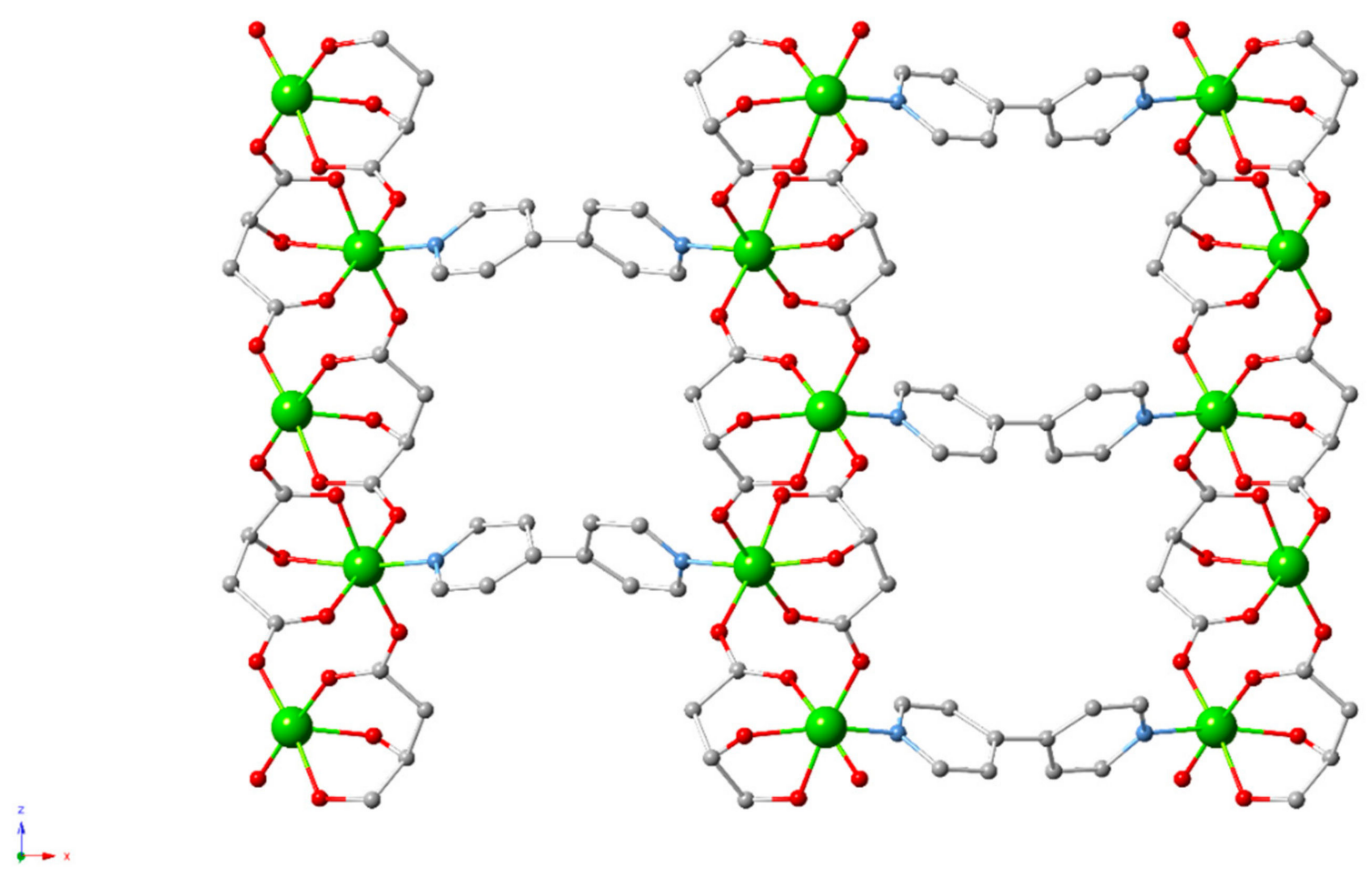
Cu and Ni-based homochiral L-malate MOFs [130].

Malate is one of the final products in the Krebs cycle and is a dicarbonyl compound. L-malic acid has been used to synthesise homochiral MOFs, involving metals such as Mn, Ni, Cu, Co and Ca [126,127,128,129,130]. In 2013 and 2014, Fedin et al. reported multiple Cu and Ni based homochiral MOFs using L-malate and N donor ligands, such as bpy (4,4′-bipyridyl) and bpe (trans-1,2-bis(4-pyridyl)ethylene) (Figure 13) [129,130]. The Cu(II)–malate layers and Ni(II)–malate chains were connected by the N-donor linkers, different lengths of which could be used to control the size of pores. A more recent application of a Cu analogue was as an HPLC separation agent for enantiomers [131].

**Figure 14:**
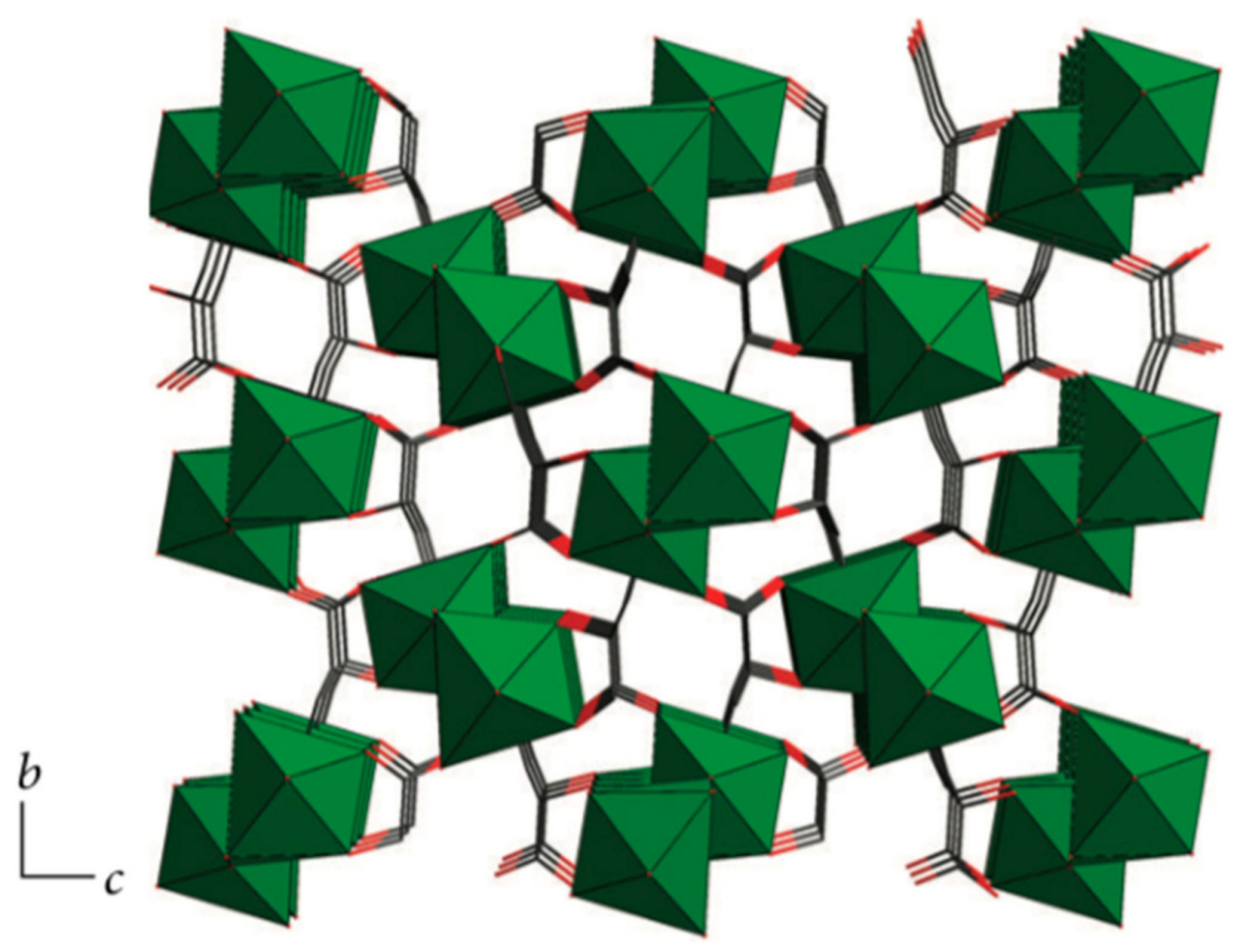
[Ca(HL^1^-MA)]_n_, a Ca and L-malic acid CP viewed along the a-axis where Ca is represented by green, C by black, O by red and H omitted for clarity. Adapted from [128]. This article is licensed under a Creative Commons Attribution 3.0 International License, which permits use, sharing, adaptation, distribution and reproduction in any medium or format, as long as appropriate credit is given to the original author(s) and the source, a link to the Creative Commons license is provided, and changes made are indicated. https://creativecommons.org/licenses/by/3.0/ This image is a cropped version of the original figure in D. S. Raja, J.-H. Luo, T.-G. Chang, S.-H. Lo, C.-Y. Wu and C.-H. Lin, J. Chem., 2013, 2013, 7.

A Ca based CP was reported in 2013 with the distinction of being a non-mixed system (Figure 14) [128]. The MOF, of the formula [Ca(HL^1^-MA)]_n_ (H_3_L^1^-MA = L-malic acid), was synthesised solvothermally and had a highly stable 3D structure, with decomposition observed above 410 °C in the thermogravimetric analysis (TGA). A biocompatible material consisting solely of Ca(II) and L-malic acid, this MOF could have potential bioapplications.

**Figure 15:**
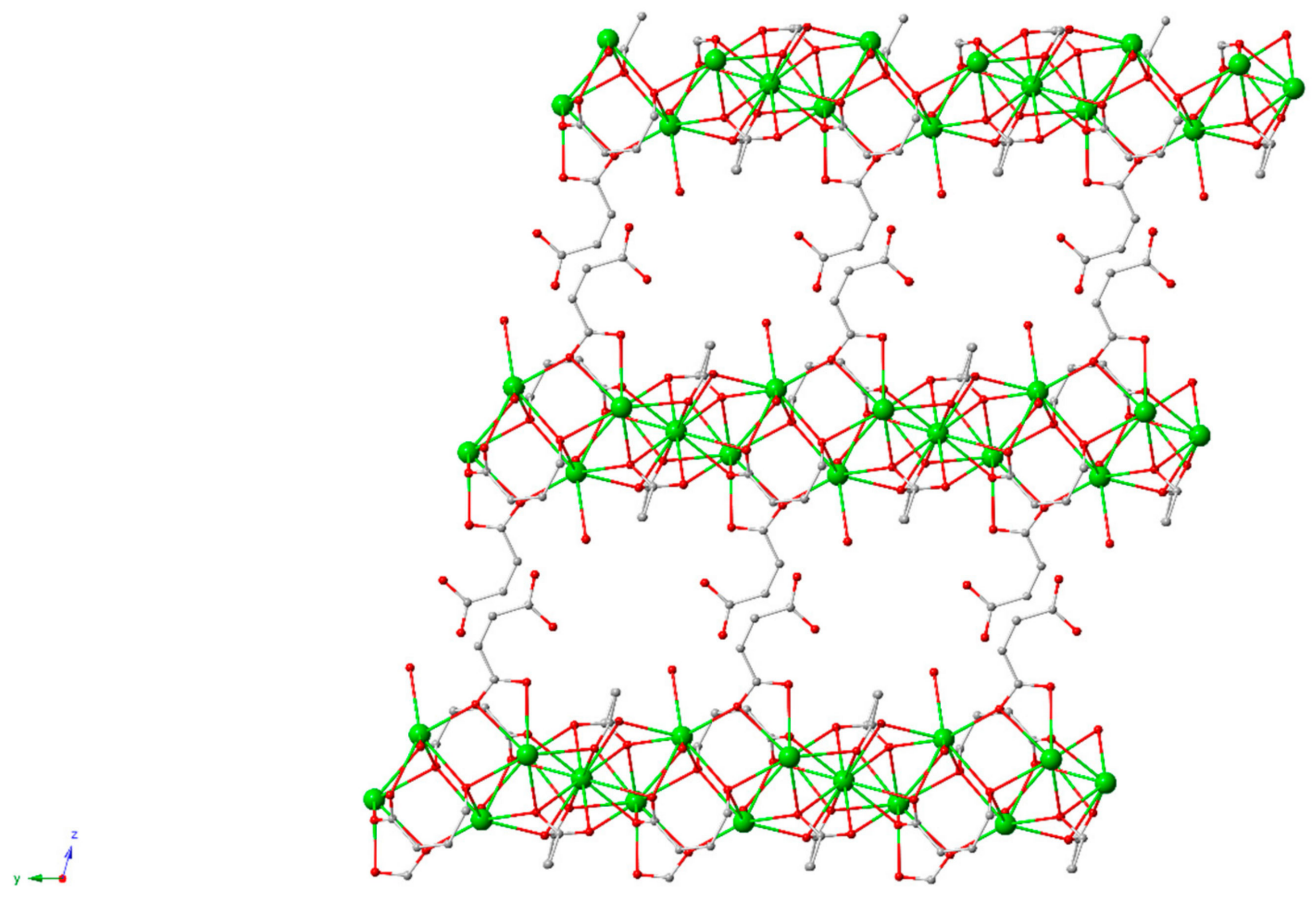
Structure of BM, a barium-maleate 2D material [132].

A 2D porous barium-maleate polymorph was reported in 2014 (Figure 15); the authors suggested it could be useful in optoelectronics or for the typical MOF applications of gas adsorption and storage, however, barium is used as an X-ray contrast medium, potentially highlighting an alternative use for this structure [132].

### 3.4. Peptides as Linkers: Biorelated Structures

Instead of requiring additional, ancillary ligands to create 3D structures, using peptides instead of amino acids can increase the complexity of the ligand and the number of functional groups available for coordination. Peptides can be used as therapeutic agents in their own right, as they naturally act as hormones and transmitters [133].

#### 3.4.1. A Conformationally Active MOF Made of and Mimicking Peptides

**Figure 16:**
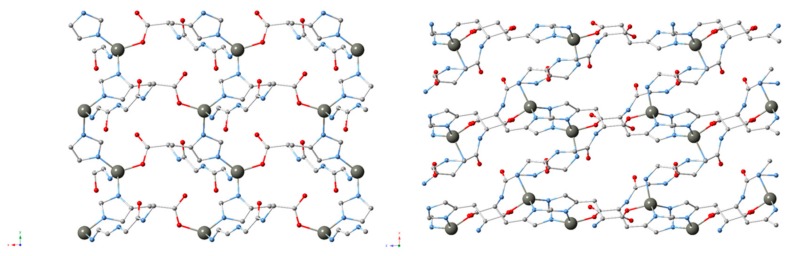

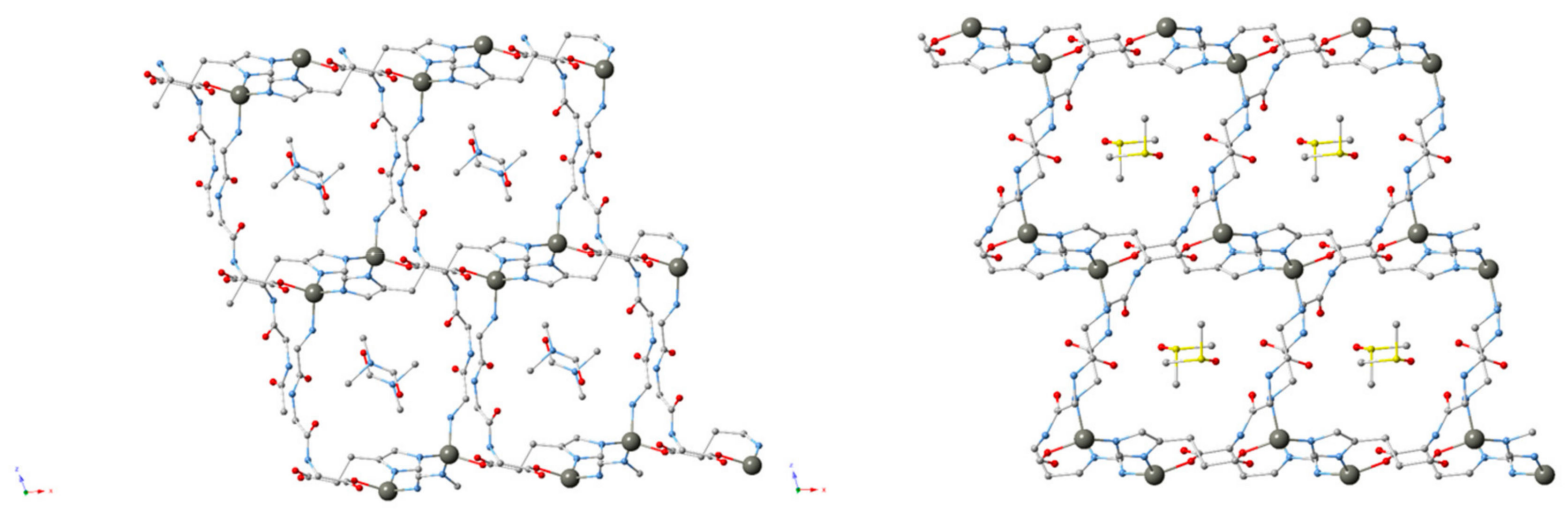
MOFs consisting of the tripeptide glycine–glycine–l-histidine (GGH), Zn and different solvents (clockwise, upper left to lower left: ZnGGH-1, -9, -2 and -3) [134].

Recently, peptides were used to create a flexible MOF, which acted similarly to free peptides which exist in multiple conformations due to rotations about the peptide chain (Figure 16) [134]. Instead of existing in one, stable conformation, nine distinct crystal structures were seen. Change in the peptide conformation could be chemically induced, resulting in a change from a conformation inactive for guest molecule sorption to one that is active.

**Scheme 12:**
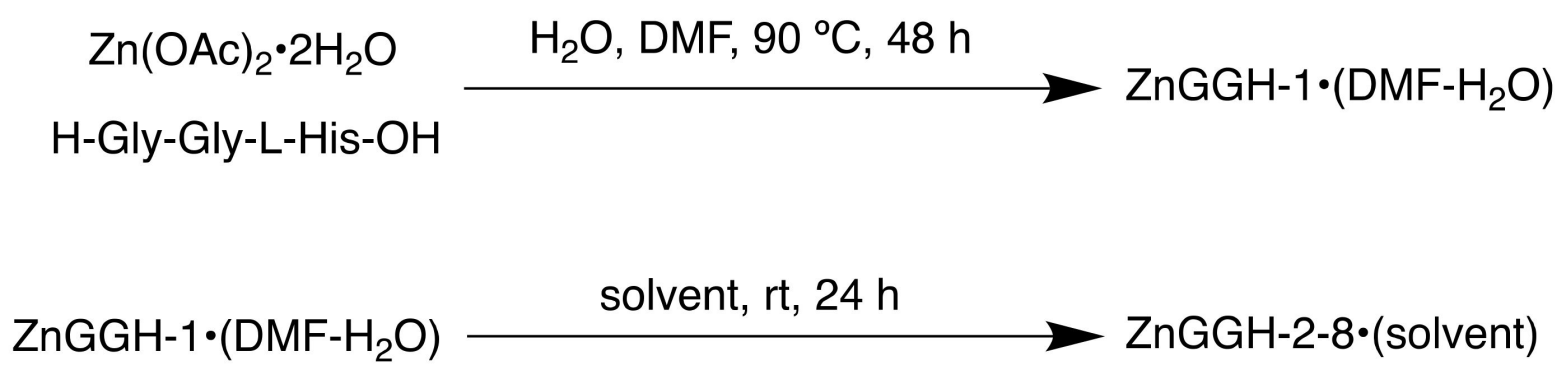
Synthesis of ZnGGH-1-8•(solvent), including DMF-H_2_O, DMSO, DMF, H_2_O, H_2_O-DMF, furfural and furfuryl alcohol. The term (solvent) in the MOF’s name denotes the solvent used in the synthesis and contained in the pores.

The MOF was synthesised solvothermally from the tripeptide glycine–glycine–l-histidine (GGH) and Zn, affording a 3D chiral MOF, ZnGGH-1 (DMF-H_2_O) (Scheme 12). Soaking the MOFs in different solvents chemically induced change; DMF and H_2_O were able to access the pores of ZnGGH-2 (DMSO) and exchange with DMSO within the pores to form the new structures ZnGGH-3 (DMF) and ZnGGH-4 (H_2_O). Crystals of eight solvated ZnGGH structures were obtained by soaking ZnGGH-1 (DMF-H_2_O) in different solvents and guests. Using density functional theory (DFT), it was found that, apart from ZnGGH-4 (H_2_O), each of the host structures in the absence of molecules within the pores converged to one of three calculated empty structures.

Solvent exchange and subsequent trigger of conformational reorganisation to one of three local minima, involving a twisted or straight linker conformation, created structures active or inactive for guest uptakes, such as dioxane or cyclopentanol. The pore geometry was modified by chemical control of the peptide linker. This study exemplifies the power peptides and their analogues can have in the synthesis of bioinspired structures, but conformational flexibility can lead to unpredictability and instability if not controllable, a potential downside to the use of such flexible molecules in MOF design.

#### 3.4.2. Pseudopeptides as Ligands, for Use in Catalysis

**Figure 17:**
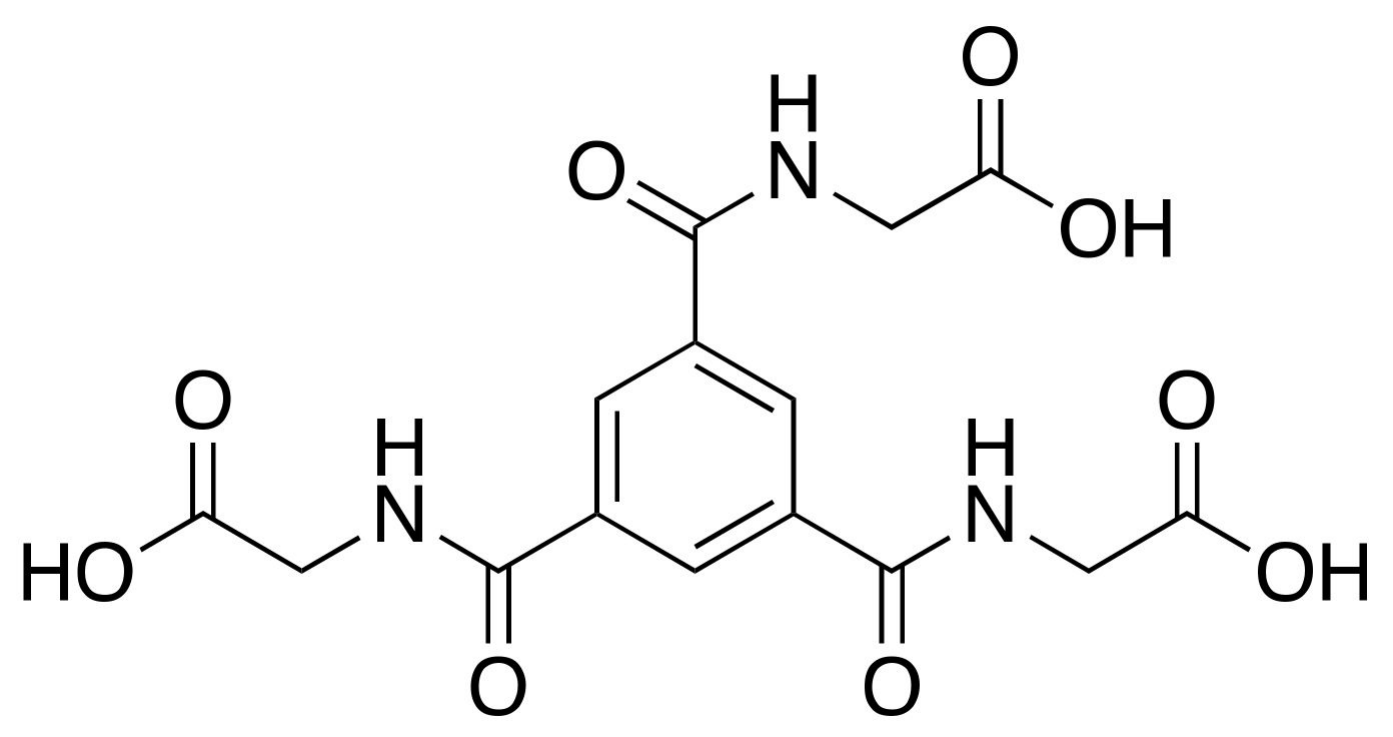
H_3_L^1^, the ligand used in the study.

In addition to peptides, pseudopeptides (backbone-modified peptides) can be used as MOF linkers. In 2018, a ThSi_2_ (ths) CP of the formula [Cu_3_(L^1^)_2_(H_2_O)_8_]·8H_2_O was reported, which was the first example of a 12-fold interpenetrated ths net [135]. The pseudopeptidic ligand, *N*,*N*,*N-*tris(carboxymethyl)-1,3,5-benzenetricarboxamide or trimesoyl-tris-glycine, was based on glycine, the simplest amino acid (Figure 17).

**Scheme 13:**
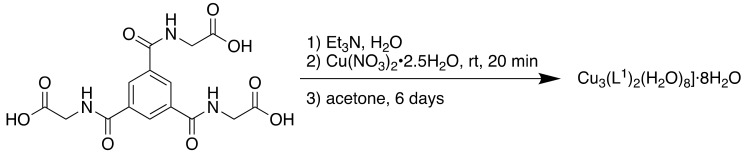
Synthesis of Cu_3_(L^1^)_2_(H_2_O)_8_]·8H_2_O.

The MOF was synthesised solvothermally followed by liquid diffusion in acetone (Scheme 13).

The MOF was tested for use as a catalyst in the A^3^ (aldehyde-alkyne-amine) coupling reaction for the synthesis of propargylamines, key intermediates in the synthesis of several N-containing biologically active compounds [136]. The compound demonstrated high activity as a heterogeneous catalyst, particularly with cyclic secondary amines and saturated aliphatic aldehydes, with yields up to 99% and a catalyst loading of 3 mol % (Table 5).

The CP lacked porous channels, and as such, it was theorised that the catalysis took place on the CP surface. This work demonstrates that pseudopeptidic ligands can be used to synthesise CPs for the development of organic catalysts.

## 4. Conclusions

Increasingly, researchers are looking beyond applications such as gas separation and fuel storage into areas including medicine [11,137]. It is clear that the possible applications of MOFs are vast, as highlighted in Table 6. These examples demonstrate the unique advantages MOFs can provide in a biomedical context. The synthesis of bioMOFs from biocompatible metals and APIs, and the resultant degradation of the material within a physiological environment offers the potential for slow release drug formulations [74], as does the more traditional encapsulation of an API within the pores of a MOF [33]. These strategies can be combined, to allow the co-delivery of drugs both as part of a framework and within the pores [81,82]. This synergistic effect can also be realised by the simultaneous action of a bioactive cation and linker, such as utilising the antimicrobial properties of zinc [34]. In addition to encapsulating traditional small molecule inhibitors, MOFs can be used to transport and deliver larger, complex molecules, such as hormones [31]. Post-synthetic modification can be a valuable tool to alter properties such as dispersion and chelation abilities [54]. Hydrophilicity and hydrophobicity are important parameters in drug design [138], and the tunable nature of MOFs, allow these to be modified as needed.

The process of developing and bringing to market a successful drug or formulation is long and expensive [139], and while significant progress has been demonstrated in terms of proof-of-concept medicinal MOFs, further studies are needed before widespread clinical use becomes a reality. While some MOF studies have demonstrated in vivo compatibility, many only demonstrate activity in vitro, and as such further, comprehensive assessments into their activity and toxicity need to be carried out. In addition, long-term biocompatibility studies will be required before more extensive clinical trials take place. This involves assessing potential degradation pathways, metabolites, target specificity and side effects, in addition to factors such as release profiles and accumulation. The multiple components of a MOF increase the complexity of these assessments; a typical small molecule inhibitor drug is one component under 500 daltons, whereas a MOF DDS could incorporate this in addition to other organic ligands and metal ions. Yet, recent research has focused on novel pharmaceutical agents with more complex behaviours, such as PROTACs and siRNA therapeutics [140,141,142]. As discussed, the use of biocompatible materials can address some of these challenges. The first clinical trial involving a MOF began only in April 2018 [20], and it is clear that while using MOFs for biomedical applications has gained popularity since Férey’s seminal work in 2006 [16], further research is required before they are fully understood. While medicinal MOFs may receive less attention than those destined for fuel storage, these porous materials have the potential to improve treatments and human health in a unique manner.

## Figures and Tables

**Table 1:** Properties of the MOFs used in the study.

MOF	Cage Size (Å)	Window Size (Å)	Hydrophobic or hydrophilic?	Notes
**MIL-100 (Fe)**	25 and 29	4.8-5.8 and 8.6	Hydrophilic	Mesoporous cages accessed through microporous windows
**MIL-127 (Fe)**	6 (channel system) 10 (cages)	6 3	Hydrophobic Hydrophilic	Two pore types; an accessible one-dimensional channel system, and hydrophilic cages accessed through narrow apertures
**UiO-66 (Zr)**	11 and 8	5–7	Slightly hydrophobic	Octahedral and tetrahedral cavities accessed through triangular micropores

**Table 2:** Brunauer–Emmett–Teller (BET) and Langmuir surface areas of M_2_(olz).

M	BET Surface Area (m^2^ g^-1^)	Langmuir Surface Area (m^2^ g^-1^)
Mg	2545	4593
Fe	1485	2618
Co	2060	3838
Ni	2067	3813
Zn	636	770

**Table 3:** Adsorption capacities of various adsorbents.

Adsorbent	Temperature	Adsorption Capacity (mg g^−1^)
Ammonia-treated CMK-3	25	769.2 [114]
Aluminium (Al_2_(SO_4_)_3_•18H_2_O)-succinic acid MOFs	25	332.5 [115]
Magellanic peat	10	168.9 [116]
Ammonia-tailored CMK-3	25	509.0 [117]
Coal fly ash	60	103.1 [118]
Pumice stone	25	72.5 [119]
Al-SA MOF	25	739.3 [109]

**Table 4:** Adsorption isotherm parameters for the adsorption of AB1 onto Al-SA MOF and commercial PAC, where Q_max_ = predicted maximum adsorption capacity.

Adsorbents	Langmuir		
	Q_max_	k_L_	R^2^
Al-SA MOF	689.38	0.0621	0.9896
Commercial PAC	168.42	0.9326	0.9622

**Table 5:** Selected results from the A^3^ coupling reaction (iPrOH, 90 °C, 24 h, air atmosphere, 3 mol % catalyst).

Entry	Aldehyde	Amine	Alkyne	Yield ^a^ (%)
**1**	Cyclohexane carboxaldehyde	Pyrrolidine	Phenylacetylene	99
**2**	Cyclohexane carboxaldehyde	Piperidine	Phenylacetylene	99
**3**	Cyclohexane carboxaldehyde	Diethylamine	Phenylacetylene	77
**4**	Benzaldehyde	Pyrrolidine	Phenylacetylene	67
**5**	Cyclohexane carboxaldehyde	Pyrrolidine	1-hexyne	95
^a^ NMR yields based on aldehyde				

**Table 6:** A summary of the MOFs discussed, with bioapplications and with bioinspired design.

Entry	Compound Name	Formula or Formulation	Applications
**MOFs with bioapplications**			
**1**	MIL-100 (Fe)	[Fe_3_O(H_2_O)_2_OH(C_9_H_3_O_6_)_2_]·*n*H_2_O	Drug delivery (aspirin and ibuprofen) [33]
**2**	MIL-127 (Fe)	[Fe_3_O(OH)_0.88_Cl_0.12_(C_16_N_2_O_8_H_6_)_1.5_(H_2_O)_3_]·*n*H_2_O	
**3**	UiO-66 (Zr)	[Zr_6_O_4_(OH)_4_(C_8_O_4_H_4_)_6_]·*n*H_2_O	
**4**	CD-MOF-1	[(C_48_H_80_O_40_)(KOH)_2_]_n_	Drug delivery (ibuprofen) [40]
**5**	Hf_6_-DBA	Hf_6_(μ_3_-O)_4_(DBA)_6_	Radioenhancers [48]
**6**	Hf_12_-DBA	Hf_12_(μ_3_-O)_8_(μ_3_-OH)_8_(μ_2_-O)_6_(DBA)_9_	
**7**	MIL-100 (Fe)	MCM@PEG-CO-DOX	CO therapy, drug delivery (Doxorubicin) [54]
**8**	MIL-127 (Fe)	Fe_3_(OH)_0.66_Cl_0.33_(C_16_N_2_O_8_H_6_)_1.5_(H_2_O)_3_·nH_2_O	Detoxifying agent (aspirin) [56]
**9**	bio-MOF-11-Co	Co_2_(adenine)_2_(CO_2_CH_3_)_2_·2DMF·0.5H_2_O	Paraben degradation catalyst [68]
**Bioinspired MOFs**			
**10**	Mg_2_(olz)	Mg_2_(C_14_H_10_N_2_O_6_)	Drug delivery/co-delivery (PEA) [81]
**11**	medi-MOF-1	[Zn_3_(C_21_H_20_O_6_)_2_·7(DMA)·3(EtOH)]	Drug delivery/co-delivery (ibuprofen) [82]
**12**	Mg-nalidixic acid	C_12_H_17_N_3_O_9_Mg	Antibacterial agents [89]
**13**	UTSA-16	[KCo_3_(C_6_H_4_O_7_)(C_6_H_5_O_7_)(H_2_O)_2_]·8H_2_O	Oxygen evolution reaction (OER) electrocatalyst [100]
**14**	MOF-1201	Ca_14_(L-lactate)_20_(acetate)_8_(C_2_H_5_OH)(H_2_O)	Fumigant sorption formulation [105]
**15**	MOF-1203	Ca_6_(L-lactate)_3_(acetate)_9_(H_2_O)	
**16**	Al-SA	-	Azo dye adsorption material [109]
**17**	MOF-801	Zr_6_O_4_(OH)_4_(fumarate)_6_	Fluoride adsorption material to treat brick tea [121]
**18**	CaFu	-	
**19**	BM	C_24_H_14_O_24_Ba_5_⋅7H_2_O	Gas storage and separation, optoelectronics [132]
**20**	ZnGGH-1-9	Zn(glycine–glycine–L-histidine)-n•(solvent)	Guest molecule sorption [134]
**21**	ThSi_2_ CP	[Cu_3_(L^1^)_2_(H_2_O)_8_]·8H_2_O	A^3^ coupling catalyst [135]
